# Insights into solvent and surface charge effects on Volmer step kinetics on Pt (111)

**DOI:** 10.1038/s41467-023-37935-6

**Published:** 2023-04-25

**Authors:** Jon C. Wilson, Stavros Caratzoulas, Dionisios G. Vlachos, Yushan Yan

**Affiliations:** 1 Department of Chemical and Biological Engineering, University of Delaware, 150 Academy St, Newark, DE 19716 USA; 2 Catalysis Center for Energy Innovation, University of Delaware, 221 Academy St, Newark, DE 19716 USA

**Keywords:** Energy, Electrochemistry, Catalytic mechanisms, Reaction mechanisms

## Abstract

The mechanism of pH-dependent hydrogen oxidation and evolution kinetics is still a matter of significant debate. To make progress, we study the Volmer step kinetics on platinum (111) using classical molecular dynamics simulations with an embedded Anderson-Newns Hamiltonian for the redox process and constant potential electrodes. We investigate how negative electrode electrostatic potential affects Volmer step kinetics. We find that the redox solvent reorganization energy is insensitive to changes in interfacial field strength. The negatively charged surface attracts adsorbed H as well as H^+^, increasing hydrogen binding energy, but also trapping H^+^ in the double layer. While more negative electrostatic potential in the double layer accelerates the oxidation charge transfer, it becomes difficult for the proton to move to the bulk. Conversely, reduction becomes more difficult because the transition state occurs farther from equilibrium solvation polarization. Our results help to clarify how the charged surface plays a role in hydrogen electrocatalysis kinetics.

## Abstract (web summary)

Excess free charges on electrode surfaces drive changes in hydrogen electrocatalysis kinetics. Here, the authors show how redox solvent reorganization energy is insensitive to interfacial electric field strength; instead, the charged surface directly modulates proton electrochemical potential.

## Introduction

Designing efficient and cost-effective electrochemical devices necessitates a deep understanding of the electrochemical interface where there is a rich interplay of physical phenomena. Historically, the study of chemisorption on electrodes has generated fruitful links between the electronic structure of catalysts and their electrocatalytic activity^1,2^. The resulting linear scaling relationships from these works have been useful guides for catalyst design; for example, hydrogen binding energy (HBE) correlates well with catalyst activity across several orders of magnitude^3–5^. However, emerging research indicates a more detailed microscopic understanding of heterogeneous electron transfer (ET) is needed to resolve ongoing debates about kinetic trends^6^. We are particularly interested in understanding the origin of sluggish alkaline hydrogen oxidation and evolution (HOR/HER) kinetics. At its simplest, the reaction for HOR/HER can be written as follows in acid and base, respectively:

1 $${H}_{2}\leftrightarrow 2{H}^{+}+2{e}^{-}$$

2 $${H}_{2}+2O{H}^{-}\leftrightarrow 2{H}_{2}O+2{e}^{-}$$

In base, the exchange current density for platinum group metals (PGMs) is about 2 orders of magnitude smaller than in acid. Changes in HBE and water structure, interfacial field strength, cation, and hydroxyl adsorption, and variation in HOR/HER mechanism have all been invoked in explanations for pH dependence of HOR/HER activity^3,7–12^. These theories have been compared extensively in reviews by others^6,13,14^. Here, our goal is to understand how the charged surface and double layer electrostatic potential affect HOR/HER kinetics.

Recently, there has been increasing interest in the potential of zero free charge (PZFC) of catalysts as a descriptor for pH dependent HOR/HER kinetics. As pH is increased, the equilibrium potential for HOR/HER shifts negatively by 59 mV/pH unit. However, on Pt (111), the PZFC is about 0.3V vs. SHE and is relatively constant with pH. Following the Nernstian shift in equilibrium potential, the alkaline working potential is farther below the PZFC and thus the electrode electrostatic potential is more negative, altering the electrochemical double layer (EDL) structure and composition. Such EDL effects are most relevant within around $1{{{{{\rm{nm}}}}}}$ of the electrode surface where the local field can exceed $1{{{{{\rm{V}}}}}}/{{{{{\rm{nm}}}}}}$ and the local concentration of electrolyte species can deviate from the bulk concentration by multiple orders of magnitude. Thus far, mechanistic explanations linking such EDL effects to HOR/HER kinetics have been difficult to establish conclusively^6^.

To frame how we study the effect of EDL electrostatics on HOR/HER, we briefly recap the possible mechanisms by which the charged electrode surface can affect redox kinetics. In the 1930’s, A.N Frumkin described how the local potential near the surface affects the kinetics for heterogeneous charge transfer^15,16^. When the electrode surface has nonzero free surface charge, the resulting electrostatic potential decays over some distance from the surface. The double layer electrostatic potential arising from the charged surface alters the electrochemical potential of double layer ions. Consequently, ions of unlike/like charge accumulate/deplete in the double layer, and the free energy change and activation energy associated with redox reactions are modulated. These electrostatic effects on kinetics are often called the “Frumkin effect” in the literature.

**Fig. 1:**
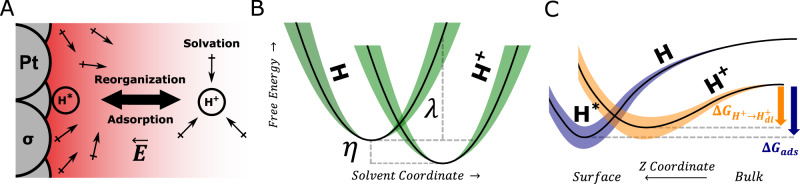
Schematics of Volmer reaction along the collective solvent coordinate and proton-surface distance, z. **A** Illustration of chemisorption and solvent reorganization around ${{{{{{\rm{H}}}}}}}^{+}$ in the Volmer step at charged Pt (111). Approaching the electrode, ${{{{{{\rm{H}}}}}}}^{+}$ moves through the interfacial field and loses significant solvation energy before reaching the charge transfer transition state. **B** Diabatic free energy curves of the ${{{{{{\rm{H}}}}}}}^{+}/{{{{{\rm{H}}}}}}$ pair at fixed z; solvent reorganization energy, *λ*, and overpotential, *η*, are shown. Changes in the EDL field strength can potentially modulate solvent polarization fluctuations, which in turn determines the free energy curvature (green) and solvent reorganization energy. **C** Free energies of the ${{{{{{\rm{H}}}}}}}^{+}/{{{{{\rm{H}}}}}}$ pair projected onto the z coordinate. Reduced hydrogen at the surface is denoted ${{{{{{\rm{H}}}}}}}^{*}$. The electrode electrostatic potential can affect the energy to move ${{{{{{\rm{H}}}}}}}^{+}$ to the double layer, ${\Delta G}_{{{{{{{\rm{H}}}}}}}^{+}\to {{{{{{\rm{H}}}}}}}_{{{{{{\rm{dl}}}}}}}^{+}}$, by changes in double layer electrostatics and solvation. In the adiabatic representation, ${{{{{{\rm{H}}}}}}}^{*}$ has a partial charge, and thus HBE is also affected by electrostatics. More broadly, the change in double layer structure and composition with changing surface potential can also indirectly affect the free energy of hydrogen near the surface.

Beyond the direct electrostatic effect on ions, the interfacial electric field also polarizes the solvent, which may affect redox kinetics. Such electric field effects are complicated by the tendency for water to order at the surface. For example, at the catalyst surface, favorable platinum-water interactions induce water molecules to form a structured hydrogen bonding network. Moreover, the interfacial region has a reduced dielectric constant owing to slow orientational relaxation. In Ledezma-Yanez’s view, the water network becomes even more rigid as the interfacial field strength increases, impeding motion of charged species through the double layer, slowing alkaline HER kinetics^17^. On the other hand, based on dielectric continuum theory, the reorganization energy for electron transfer should diminish when strong interfacial fields induce dielectric saturation, decreasing the redox activation barrier^18^. In Fig. 1, we summarize these perspectives schematically and conceptualize the reaction as proceeding along two reaction coordinates: the collective solvent coordinate and the ion’s distance from the surface, *z*. In Fig. 1B, C, we decompose the EDL effects caused by the charged surface onto the two reaction coordinates. The Volmer step proceeds on the 2D surface, and trends in activation energy are governed by changes in free energies with respect to both coupled coordinates. In this work, we measure both how changes in the electrode electrostatic potential directly affects the electrostatics of the redox pair ${{{{{{\rm{H}}}}}}}^{+}/{{{{{\rm{H}}}}}}$ as well as how it affects double layer solvent structure and reorganization.

To make progress in understanding EDL effects, we model the relevant physics of electron transfer and chemisorption with explicit solvent. Following the ideas of Marcus and Hush^19,20^, we model solvent dynamics along the collective solvent coordinate, using classical molecular dynamics (MD) to collect solvent fluctuation statistics. We embed the Anderson-Newns Hamiltonian (ANH) to model charge transfer while additionally incorporating controllable electrode potential into this framework for the first time^21,22^. Adding controllable electrode potential to the model enables us to study the relationship between electrode electrostatic potential and charge transfer kinetics. Moreover, explicitly modeling the electrolyte atoms allows us to probe the microscopic structure and dynamics of the EDL.

Here we show how the electrostatic potential in the EDL affects the physics of the Volmer step. In this work, we model Volmer step at the Pt (111)/water interface as a prototypical case study. To that end, we perform importance sampling along the collective solvent coordinate to bias the system to sample redox events for the electronically adiabatic reaction and then estimate redox activation energies from potentials of mean force. We show that solvent reorganization energy is insensitive to the interfacial field at physically relevant potentials. We further show how, instead, the negative electrostatic potential directly affects the kinetic barriers for the redox process by stabilization of the ${{{{{{\rm{H}}}}}}}^{+}$ state near the surface.

## Results

### Diabatic free energy curves for HER/HOR

**Fig. 2:**
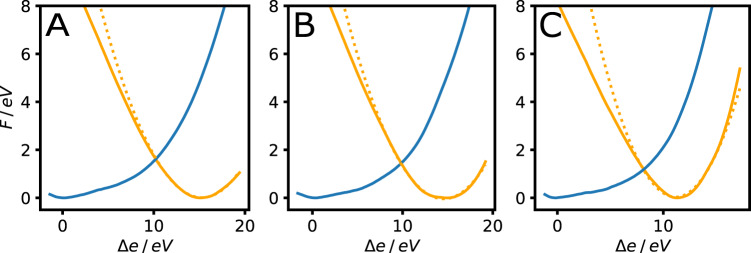
Diabatic free energies for **H**^+^ and reduced H along the solvent coordinate at fixed z. The reduced state free energies are calculated from the oxidized free energies by the relation ${F}^{{{{{{\rm{H}}}}}}}\left(\Delta e\right)={F}^{{{{{{{\rm{H}}}}}}}^{+}}\left(\Delta e\right)+\Delta e+{\epsilon }_{a}-{\epsilon }_{f}$ and the overpotential is set to $\eta=0$ in each case for ease of comparison. **A** z = 4.0 Å, **B** z = 3.0 Å, **C** z = 2.0 Å. The cell electrostatic potential is set to $V-{V}_{{{{{{\rm{pzc}}}}}}}=0{{{{{\rm{V}}}}}},$ i.e., the PZC for both electrodes. The orange and blue curves correspond to the oxidized and reduced states, respectively. Parabolas fitted near the proton minimum are shown as dashed lines.

We use the standard electron-transfer solvent collective coordinate, $\Delta E$, defined by the difference in potential energy between the redox ion’s reduced and oxidized states at a fixed nuclear configuration, i.e., the vertical energy gap^23^:

3 $$\Delta E({{{{{\bf{R}}}}}})={{{{{{\mathcal{V}}}}}}}^{{{{{{\rm{red}}}}}}}\left({{{{{\bf{R}}}}}}\right)-{{{{{{\mathcal{V}}}}}}}^{{{{{{\rm{ox}}}}}}}({{{{{\bf{R}}}}}})$$

where **R** is the set of all nuclear coordinates of the system at a given time and ${{{{{{\mathcal{V}}}}}}}^{{{{{{\rm{red}}}}}}}$, ${{{{{{\mathcal{V}}}}}}}^{{{{{{\rm{ox}}}}}}}$ are the energies of the reduced and oxidized states given by the sum of interaction potential contributions between the ion and the system. To estimate solvent reorganization energy, we first compute the diabatic free energy curves when the electrode is at the PZFC (hereafter shortened to PZC), shown in Fig. 2. (Diabatic surfaces correspond to electronic states whose character, neutral H or H^+^, does not change with molecular geometry—the solvent polarization specifically—and electron transfer implies that the system has crossed from one diabatic potential to the other.) The diabatic redox overpotential at fixed *z* is related to the Fermi level of the metal by the energy conservation relation^21,24–26^:

4 $$\eta={\epsilon }_{a}-{\epsilon }_{f}+\Delta {e}_{{{\min }}}^{{{{{{{\rm{H}}}}}}}^{+}}-\lambda$$

where ${{{{{{\rm{\epsilon }}}}}}}_{{{{{{\rm{a}}}}}}}$ is the proton’s vacuum level, ${\epsilon }_{f}$ is the Fermi level, $\Delta {e}_{{{\min }}}^{{{{{{{\rm{H}}}}}}}^{+}}$ is at the minimum of the proton diabatic free energy, i.e., equilibrium solvation, and $\lambda$ is the solvent reorganization energy. Note that specific values of the random variable $\Delta E$ are denoted by $\Delta e$. The solvent reorganization energy is calculated to be 5.5 eV in bulk, higher than previous estimates of 3-4 eV^27^. The reorganization energy decreases to 3.2 eV at $z=2.0\,{{{{{\text{\AA }}}}}}$.

In Fig. 2, moving toward the surface, $\Delta {e}_{{{\min }}}^{{{{{{{\rm{H}}}}}}}^{+}}$ decreases due to proton desolvation with a concomitant decrease in *λ*. In other words, $\Delta e$ decreases when the proton potential energy becomes less negative. The free energy curves markedly deviate from the symmetric parabolic behavior of Marcus theory, which indicates the solvent polarization does not respond linearly over the whole range. In addition, the curvature of the neutral state’s (H atom) free energy profile is smaller curvature than the charged state. As the curvature is inversely proportional to ${{\langle }}{\left(\delta \Delta E\right)}^{2}{{\rangle }}$ (the variance in the solvent collective variable, $\Delta E$), its smaller value is a manifestation of the loose solvent organization around the neutral H atom^23^. In contrast, the strong field around the ${{{{{{\rm{H}}}}}}}^{+}$ strongly polarizes its solvation water.

**Fig. 3:**
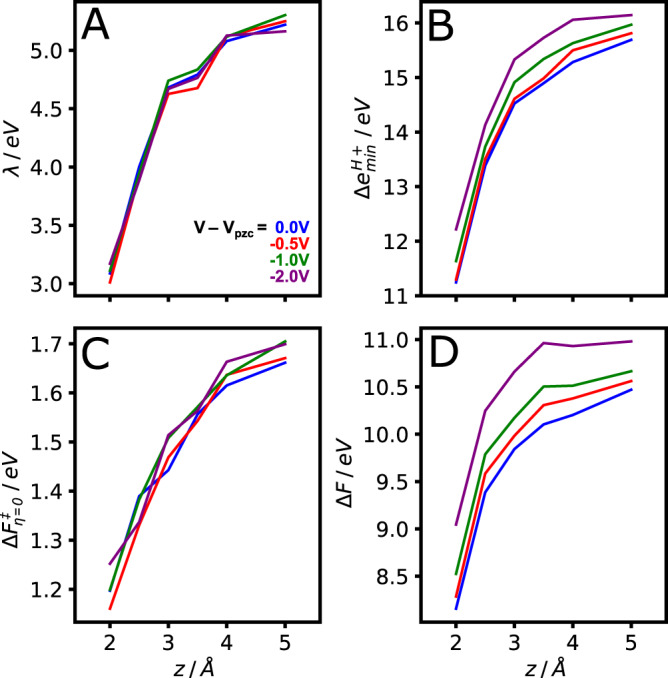
Effect of cell potential on diabatic free energies at fixed *z*. **A** solvent reorganization λ; **B** equilibrium $\Delta e$ value for ${{{{{{\rm{H}}}}}}}^{+}$, $\Delta {e}_{{{\min }}\,}^{{{{{{{\rm{H}}}}}}}^{+}};$ **C** free energy to reach the diabatic crossing with the effect of overpotential subtracted off, $\Delta {F}_{\eta=0\,}^{{{\ddagger}} }$; and **D** reduction free energy change, $\Delta F$, for the diabatic curves where ${F}^{{{{{{\rm{H}}}}}}}\left(\Delta e\right)={F}^{{{{{{{\rm{H}}}}}}}^{+}}\left(\Delta e\right)+\Delta e$. The blue, red, green, and purple curves are computed from diabatic free energy curves at electrode voltages relative to PZC of $V-{V}_{{{{{{\rm{pzc}}}}}}}=0{{{{{\rm{V}}}}}},-0.5{{{{{\rm{V}}}}}},-1{{{{{\rm{V}}}}}},-2{{{{{\rm{V}}}}}}$, respectively.

Next, we vary the electrode potential *V* and repeat the calculations presented in Fig. 2 for fixed *z* values in order to explore how the electric field of the electrode affects the diabatic free energy profiles. We have simulated a capacitor cell filled with electrolyte and control the overall cell potential, $\Delta V$. In Supplementary Note 2, we establish the relationship between cell potential, single electrode potentials, and surface charge. The potential of the electrode near H is defined on an absolute scale such that $V-{V}_{{{{{{\rm{pzc}}}}}}}=-\Delta V/2$. Figure 3A shows that the monotonic decrease in *λ* with decreasing z persists at non-zero electrode potentials, once again, on account of the proton being progressively desolvated as it approaches the surface. More interestingly, at fixed values of *z* the variation in $\lambda$ with $V$ is statistically insignificant. Correspondingly in Fig. 3C, there is no obvious trend in the intrinsic barrier. The electrode’s field (~0.1 V/Å) is not strong enough to disrupt the polarization of the solvent experiencing the proton’s strong field (>1 V/Å within 3 Å). The very limited effect of the electrode field on the reorganization energy, even at very short distances from the surface, is manifested in the unchanging equilibrium distribution of the orientation of water molecules in the proton’s solvation shell (see Supplementary Note 3), indicating that the solvent polarization is also unchanged.

As the surface is negatively charged, nearby ${{{{{{\rm{H}}}}}}}^{+}$ experiences an increasingly more negative potential, increasing $\Delta {e}_{{{\min }}}^{{{{{{{\rm{H}}}}}}}^{+}}$ and the H^+^ reduction $\Delta F$ (Fig. 3B, D). The increase in $\Delta F$ with more negative electrode electrostatic potential resembles the Frumkin effect^15^, where the driving force for redox must be corrected to include the influence of the ion’s electrostatic potential near the charged surface. The effect observed here is due to the incomplete screening of the field at the position where redox occurs. As we will show later, the ${{{{{{\rm{H}}}}}}}^{+}$ local electrostatic potential plays an important role in potential dependent kinetics.

### Potential-dependent solvent dynamics

Having established that $\lambda$ is insensitive to the interfacial field, we now explore how solvent dynamics change with field strength, which could also impact redox kinetics. Interestingly, our calculations do not support the Ledezma-Yanez view that the water network becomes more rigid with increasing interfacial field strength^17^. To measure trends in dipolar relaxation times, we calculate the normalized dipole autocorrelation function for individual water molecules as follows:

5 $${{{{{{\rm{C}}}}}}}_{{{{{{\rm{\mu }}}}}}{{{{{\rm{\mu }}}}}}}\left(t\right)=\left\langle \delta {{{{{\boldsymbol{\mu }}}}}}\left(t\right)\cdot \delta {{{{{\boldsymbol{\mu }}}}}}\left(0\right)\right\rangle /\left\langle \delta {{{{{\boldsymbol{\mu }}}}}}{\left(0\right)}^{2}\right\rangle$$

where ${{{{{\boldsymbol{\mu }}}}}}$ is the unit dipole vector of a water molecule, $\delta {{{{{\boldsymbol{\mu }}}}}}\left(t\right)$ is fluctuation from the mean, and the ensemble average is conditional over waters in a given region relative to the surface.

**Fig. 4:**
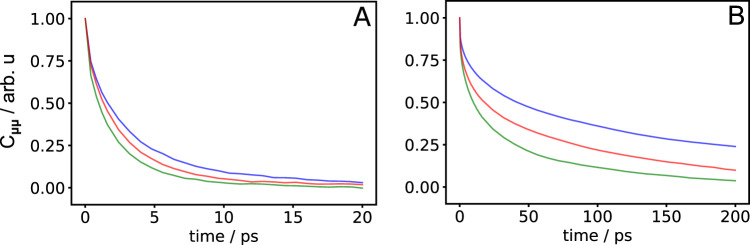
Dipole vector autocorrelation function for water molecules near the Pt (111) electrode. **A** Double layer waters near the outer Helmholtz layer within $4 \, < \, {{{{{\rm{z}}}}}} \, < \, 5\;{{{{{\text{\AA }}}}}}$. **B** Adsorbed waters where ${{{{{\rm{z}}}}}} \, < \, 3\;{{{{{\text{\AA }}}}}}$. Surface water is attracted to the Pt surface, and the lattice spacing of Pt (111) is suitable for water to form a network of hydrogen bonds parallel to the electrode plane. Consequently, the reorientation time of water increases at the surface due to confinement effects. (Blue, red, and green curves correspond to water near the electrode with voltages of $V-{V}_{{{{{{\rm{pzc}}}}}}}=0{{{{{\rm{V}}}}}},-1{{{{{\rm{V}}}}}},-2{{{{{\rm{V}}}}}}$, respectively).

In Fig. 4, we plot the conditional dipole autocorrelation function for waters around the outer Helmholtz layer and for waters adsorbed on the surface, calculated for three electrode potential values, *V* − *V*_pzc_ = 0 V, −1 V, −2 V. At short times (<1 ps), librational relaxation is dominant. At longer times, relaxation near the surface (Fig. 4B) is much slower than near the outer Helmholtz layer (Fig. 4A). Slower relaxation indicates a more sluggish solvent near the surface and concomitant decrease in the dielectric susceptibility (related to the negative of the derivative of ${{{{{{\rm{C}}}}}}}_{{{{{{\rm{\mu }}}}}}{{{{{\rm{\mu }}}}}}}\left(t\right)$^28^), which implies weaker equilibrium polarization of the surface water due to the ordering induced by the surface. At increasingly negative electrode potentials, we see increasingly faster relaxation both at the surface and in the outer Helmholtz layer and thus higher polarization. The change in polarization due to the electrode field seems to be more pronounced for surface water. Remarkably, although water retains some of its rigidity due to the presence of the surface (inferred by the lower susceptibility), the rotational mobility of the surface water seems to increase with |*V*|. It is likely that solvent dynamics accelerate at negative voltages because the interfacial field disrupts the hydrogen bond network at the surface; the average number of hydrogen bonds per water decreases as hydrogen bonding is disrupted parallel to the electrode plane (Supplementary Note 4).

We expect that the relaxation time for waters in the vicinity of the redox hydrogen are more relevant to the dynamics at the Volmer transition state in comparison to the average relaxation behavior of EDL waters. We show that the dipole relaxation of water in the proton’s solvation shell is quite insensitive to the strength of the interfacial field near the charged surface; the dynamics of waters solvating the proton are primarily determined by the strong proton field (Supplementary Note 5). We also compute the autocorrelation of the proton’s electrostatic potential (Supplementary Note 6) to estimate changes in the solvation time, and we find that solvation is somewhat faster at more negative potentials but not significantly so. Contrary to expectations, when the electrode potential is far below the PZC, the solvent network is slightly less rigid near the electrode, and proton solvation dynamics are not significantly affected by the interfacial field. Thus, another explanation must be invoked to understand how electrostatic effects due to the charged electrode are related to HOR/HER kinetic trends.

### Adiabatic free energy surfaces

To understand the interplay between chemisorption, electric potential effects, and solvent reorganization, we simulate the adiabatic free energy surfaces for the Volmer step. Close to the surface, the electronic coupling between the ion state and the surface states is large, and the reaction may be treated as electronically adiabatic. We perform importance sampling to obtain the potentials of mean force with respect to the solvent collective motion (Δ*E*) and the proton translational motion in the z-direction.

It is important to clarify the relationship between the reaction overpotential and the electrode potential in the context of this model. When the ANH is embedded into classical MD, the Fermi level is a model input parameter which determines the overpotential. The Fermi level corresponds to the real potential of the uncharged electrode which is coupled to the hydrogen ion. The applied potential difference $\Delta V$ is another independent input parameter which sets the cell potential. In a real cell, these two variables are inextricable; as the cell potential is changed, the working electrode’s overpotential and electrostatic potential vary together, connected by the electrochemical potential of electrons in the metal. The behavior of changing the electron electrochemical potential is known: at more negative applied potentials, the upward shift in Fermi level biases the reaction to reduction. In Supplementary Note 7, we show how increasing the ANH overpotential with a commensurate change in the electrode electrostatic potential biases the reaction to reduction. Here, however, we emphasize that our aim is to explore how changing the electrode electrostatic potential $V-{V}_{{{{{{\rm{pzc}}}}}}}$ affects the Volmer step, which is useful for addressing the claim that the PZC of an electrode is relevant as a kinetic descriptor. Therefore, we set a constant ANH overpotential across different cases, and we vary $\Delta V$. Effectively, we control $V-{V}_{{{{{{\rm{pzc}}}}}}}=-\Delta V/2$ for the working electrode. We set the Fermi level such that the reaction overpotential is slightly biased to adsorbed hydrogen.

**Fig. 5:**
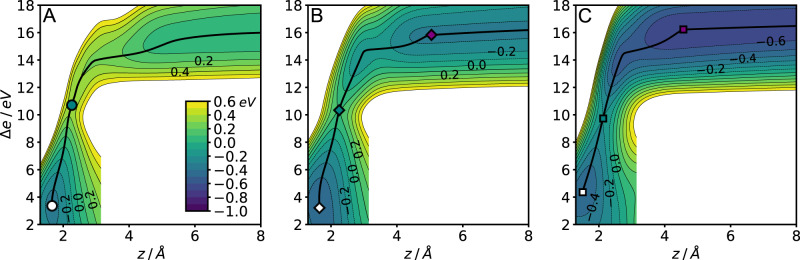
Simulated adiabatic free energy surfaces of the **H**^+^/**H** redox pair near a Pt (111) electrode as functions of the collective solvent coordinate, **Δe**, and the H’s **z**-coordinate along the normal to the surface, at varying cell electrostatic potentials. **A** $V-{V}_{{{{{{\rm{pzc}}}}}}}=-0{{{{{\rm{V}}}}}}$ **B** $V-{V}_{{{{{{\rm{pzc}}}}}}}=-1{{{{{\rm{V}}}}}}$ **C** $V-{V}_{{{{{{\rm{pzc}}}}}}}=-2{{{{{\rm{V}}}}}}$. The Fermi level is set such that ${\epsilon }_{a}-{\epsilon }_{f}=-10.19\,{eV}$. Minimum free energy paths (MFEP) are shown as traced solid lines. The white filled shapes mark the H* minima, the teal filled shapes mark the redox saddle point, ${{{{{{\rm{H}}}}}}}^{{{\ddagger}} }$, and the purple filled shapes mark the ${{{{{{\rm{H}}}}}}}_{{{{{{\rm{dl}}}}}}}^{+}$ minima at negative voltages. The hydrogen adsorption energy is calculated as the free energy difference between the H* minima and ${{{{{{\rm{H}}}}}}}^{+}$ in the bulk, which is the reference for all cases.

Figure 5 shows the adiabatic free energies for three values of the electrode potential. Approaching the surface, ${{{{{{\rm{H}}}}}}}^{+}$ is attracted by the image potential and the negative surface. Below $z=4\;{{{{{\text{\AA }}}}}}$, H^+^ becomes less coordinated to nearby waters, see Supplementary Note 8. At more negative $V-{V}_{{{{{{\rm{pzc}}}}}}}$, ${{{{{{\rm{H}}}}}}}^{+}$ becomes trapped near the surface around $z=4-5\;{{{{{\text{\AA }}}}}}$, where it experiences a significant negative potential but still retains a solvation structure similar to that of the bulk proton. In comparison to the PZC, the trapping of ${{{{{{\rm{H}}}}}}}^{+}$ in the double layer results in a later reduction transition state with concomitant increase in the reduction activation free energy. At $z \, < \, 3\;{{{{{\text{\AA }}}}}}$, most of the change along the minimum free energy pathway (MFEP) is solvent reorganization. We calculated the adsorption energies of hydrogen at this value of ${\epsilon }_{a}-{\epsilon }_{f}$ to be $-0.34\pm 0.03$ eV at $V-{V}_{{{{{{\rm{pzc}}}}}}}=0$ V, $-0.49\pm 0.03$ eV at $-1$ V, and $-0.47\pm 0.03$ eV at $-2$ V.

In Fig. 5, we connected the three cases with a constant reference state to compare the changes in free energies as $\Delta V$ is varied. Since we have not considered the presence of electrolyte salt, the solution has a long Debye length, and H^+^ is influenced by the surface field even far away. We select the constant reference state for all the adiabatic simulations as the proton at $z=25\;{{{{{\text{\AA }}}}}}$ and $V-{V}_{{{{{{\rm{pzc}}}}}}}=0$ V where it is not influenced by excess surface charge. Our choice of reference is consistent with experiment: in a macroscopic cell, protons ultimately are sourced from the bulk, i.e., where the proton activity is determined purely by its solution chemical potential. As shown in Supplementary Note 9, as the cell potential is varied, the only difference in proton free energy in the solution is due to the difference in its electrostatic potential, which is easily calculated to establish a common scale.

**Fig. 6:**
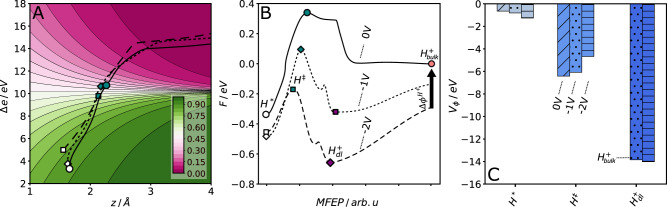
Further analysis of the adiabatic 2D surfaces at varying ***V*–*V***_**pzc**_. **A** Contours of expected H orbital occupancy ${{\langle }}{{{{{{\rm{n}}}}}}}_{{{{{{\rm{a}}}}}}}{{\rangle }}$ versus the collective solvent coordinate, $\Delta e$, and the ${{{{{\rm{z}}}}}}$-coordinate of redox H along the normal to the surface. **B** Free energy profiles along respective MFEPs of adiabatic free energy surfaces shown previously in Fig. 4. **C** Average electrostatic potential energy ${V}_{\phi }={q}_{H}\left\langle \phi \right\rangle$ experienced by ${{{{{{\rm{H}}}}}}}^{*},$ ${{{{{{\rm{H}}}}}}}^{{{\ddagger}} }$, ${{{{{{\rm{H}}}}}}}_{{{{{{\rm{dl}}}}}}}^{+}$ at the three applied potentials. The partial charge of hydrogen is related to its occupancy by ${q}_{H}=1-\langle {n}_{a}\rangle$. **A** The overlaid solid, dotted, and dashed lines are the minimum free energy paths for the free energy surfaces corresponding to $V-{V}_{{{{{{\rm{pzc}}}}}}}=0{{{{{\rm{V}}}}}},-1{{{{{\rm{V}}}}}},-2{{{{{\rm{V}}}}}}$, respectively. **B** White shapes: Adsorbed H (H*). Teal shapes: Redox transition states (${{{{{{\rm{H}}}}}}}^{{{\ddagger}} }$). Purple shapes: Proton minima in the double layer (${{{{{{\rm{H}}}}}}}_{{{{{{\rm{dl}}}}}}}^{+}$). Pink circle: bulk proton reference state (${{{{{{\rm{H}}}}}}}_{{{{{{\rm{bulk}}}}}}}^{+}$). **C** the bars correspond to 0 V (diagonal hatches), −1 V (dots), and −2 V (horizontal hatches). ${V}_{\phi }$ for the reference, ${{{{{{\rm{H}}}}}}}_{{{{{{\rm{bulk}}}}}}}^{+}$, is shown for comparison to ${{{{{{\rm{H}}}}}}}_{{{{{{\rm{dl}}}}}}}^{+}$.

The changes in hydrogen binding energy can be understood in terms of the expected orbital occupancy ${{\langle }}{n}_{a}{{\rangle }}$ of H* and its electrostatic attraction to the surface. In Fig. 6A, we show how ${{\langle }}{n}_{a}{{\rangle }}$ changes along the MFEPs for the adiabatic surfaces shown in Fig. 5. The equilibrium $\Delta {{{{{\rm{E}}}}}}$ value for ${{{{{{\rm{H}}}}}}}^{*}$, $\Delta { e }_{{{\min }}}^{{{{{{{\rm{H}}}}}}}^{*}},$ is still significantly positive, due to the stabilization of the oxidized state by water and the polarizable surface. Consequently, the oxidized state is more favored in comparison to adsorbed H in the absence of solvent, and ${{\langle }}{n}_{a}\rangle$ ranges from 0.8-0.9. The expected occupancy of H* determines its partial positive charge, which takes a value of ${q}_{H}=1-\left\langle {n}_{a}\right\rangle$. At $V-{V}_{{{{{{\rm{pzc}}}}}}}=0{{{{{\rm{V}}}}}}$, ${{{{{{\rm{q}}}}}}}_{{{{{{\rm{H}}}}}}}$ for H* is about $+0.12$, increasing to $+0.16$ at $V-{V}_{{{{{{\rm{pzc}}}}}}}=-2{{{{{\rm{V}}}}}}$. Furthermore, going from $V-{V}_{{{{{{\rm{pzc}}}}}}}=0{{{{{\rm{V}}}}}}$ to $-1{{{{{\rm{V}}}}}}$ and to $-2{{{{{\rm{V}}}}}}$, H* experiences a more negative electrostatic potential due to the negative surface. From Fig. 6C, the electrostatic potential energy of H* correspondingly becomes more negative, and consequently binding energy increases. While H adsorption becomes more favorable by $0.15$ eV going from $0{{{{{\rm{V}}}}}}$ to $-1{{{{{\rm{V}}}}}}$, there is no similar increase as the potential is further lowered to $-2{{{{{\rm{V}}}}}}$. At very negative potentials, the stronger electrostatic attraction to the surface is offset by a weaker Pt-H bond as ${{\langle }}{n}_{a}\rangle$ for H* decreases.

The experimental voltametric feature for H adsorption on Pt (111) is quite broad and we cannot directly compare our theoretical HBE trends to experiment without a model of coverage effects. We also cannot attempt to explain pH-dependent hydrogen electrosorption peak shifts with this argument due to the neglect of electronic structure changes that accompany changes in absolute electrode potential^29^. However, because the change in HBE observed here is likely due to the negative electrostatic potential at the surface, we hypothesize that shifts in the PZC induced by surface adsorbates can cause non-Nernstian shifts in HBE. In the future, grand-canonical DFT calculations of hydrogen-metal electronic interactions with explicit solvation may be useful to estimate the partial charge of ${{{{{{\rm{H}}}}}}}^{*}$ with greater accuracy^29^.

We observe that the oxidation event at $V-{V}_{{{{{{\rm{pzc}}}}}}}=-2{{{{{\rm{V}}}}}}$ is more facile than at the PZC, however, ${{{{{{\rm{H}}}}}}}_{{{{{{\rm{dl}}}}}}}^{+}$ cannot easily escape the double layer, slowing HOR kinetics. In Fig. 6B, we plot the free energy profiles along the MFEPs of the adiabatic surfaces in Fig. 5; they track the progress of the reaction from surface ${{{{{{\rm{H}}}}}}}^{*}$ to the bulk solution ${{{{{{\rm{H}}}}}}}^{+}$ at $z=25\;{{{{{\text{\AA }}}}}}.$ The oxidation activation barrier decreases from 0.67eV to 0.58eV to 0.29eV going from $V-{V}_{{{{{{\rm{pzc}}}}}}}=0{{{{{\rm{V}}}}}}$ to $-1{{{{{\rm{V}}}}}}$ and to $-2{{{{{\rm{V}}}}}}$, respectively. However, in $V-{V}_{{{{{{\rm{pzc}}}}}}}=-1{{{{{\rm{V}}}}}}$ case, there is a barrier of 0.31 eV for the proton to move from the double layer around $5\;{{{{{\text{\AA }}}}}}$ to the bulk. In the $V-{V}_{{{{{{\rm{pzc}}}}}}}=-2{{{{{\rm{V}}}}}}$ case, that barrier increases to 0.68 eV. In the double layer free energy well, the H atom is proton-like in character and $\left\langle {n}_{a}\right\rangle \cong 0.005$, thus the partial charge of ${{{{{{\rm{H}}}}}}}_{{{{{{\rm{dl}}}}}}}^{+}$ is ${q}_{H}\cong+.995$ and ${{{{{{\rm{H}}}}}}}_{{{{{{\rm{dl}}}}}}}^{+}$ is attracted to the negative surface. In the present case of dilute solution, there are insufficient electrolyte ions to screen the electrostatic potential at 5–6 ${{{{{\text{\AA }}}}}}$ from the surface. However, in cases where the ionic strength is high, the negative EDL electrostatic potential is expected to concentrate cations in the double layer region, which will screen the charged surface and diminish the depth of the observed proton free energy well.

At more negative $V-{V}_{{{{{{\rm{pzc}}}}}}}$, the reduction activation energy rises due to the increase in reduction $\Delta {{{{{\rm{\it{F}}}}}}}$. At $V-{V}_{{{{{{\rm{pzc}}}}}}}=-2{{{{{\rm{V}}}}}}$, the reduction activation energy is about 0.15eV larger than at the PZC. It is seemingly counterintuitive that reduction becomes more difficult, since the ${{{{{{\rm{H}}}}}}}^{+}$ should be attracted towards the negatively charged surface where reduction occurs– in this case, the Frumkin effect would predict an increase in reduction kinetics, rather than a decrease. However, there is significant coupling between the two reaction coordinates, that is, we must consider how the solvent reorganizes as the proton proceeds toward the surface. In agreement with Marcus–Hush theory (and with Hammond’s postulate), the reduction transition state shifts towards the H* state along the solvent coordinate as the reduction $\Delta {{{{{\rm{\it{F}}}}}}}$ increases. At the saddle point ${{{{{{\rm{H}}}}}}}^{{{\ddagger}} }$, $\left\langle {n}_{a}\right\rangle$ is 0.42 at $0{{{{{\rm{V}}}}}}$ and increases to $0.57$ at $-2{{{{{\rm{V}}}}}}$, indicating an increase in the reduction charge transfer coefficient, consistent with a later transition state. At the transition state, the solvent’s inertial (mainly rotational) polarization is farther out of equilibrium with respect to solvated ${{{{{{\rm{H}}}}}}}^{+}$ when the surface is more negative, increasing the reduction activation energy. In Fig. 6C, ${V}_{\phi }$ at ${{{{{{\rm{H}}}}}}}^{{{\ddagger}} }$ is increasingly less negative relative to ${{{{{{\rm{H}}}}}}}_{{{{{{\rm{dl}}}}}}}^{+}$ as $V-{V}_{{{{{{\rm{pzc}}}}}}}$ is set more negatively, consistent with a decrease in solvation energy at the TS. Changes in the coordination numbers of water atoms around ${{{{{{\rm{H}}}}}}}^{{{\ddagger}} }$ further confirm weaker solvation at negative voltages (Supplementary Note 10). Earlier, we observed that $\lambda$ is unaffected by V. $\lambda$ is a measure of the intrinsic redox barrier, proportional to the equilibrium polarization difference between the two states. To be clear, near more negatively charged electrodes, the TS occurs farther from equilibrium H^+^ solvation due to the increase in $\Delta F,$ not a change in the intrinsic barrier.

## Discussion

So far, we have studied the electrosorption of hydrogen at the Pt (111) surface, often called the acidic Volmer step. However, to make the direct comparison to experimental results for alkaline HOR/HER, we must also carefully consider how hydroxide participates in the reaction. While we have not simulated ${{{{{\rm{O}}}}}}{{{{{{\rm{H}}}}}}}^{-}$ here, our insights from the electrostatics and solvent reorganization data could clarify existing debates.

Recently, there has been considerable debate regarding the importance of interfacial electric fields and hydroxide binding as descriptors for alkaline HOR/HER kinetics. For example, it is unclear whether ${{{{{\rm{Ni}}}}}}{\left({{{{{\rm{OH}}}}}}\right)}_{2}$ promotes alkaline HER by shifting the PZC or by a bifunctional effect, or possibly even both. Several authors have argued that alkaline HER is rate limited by water splitting at the interface since there is very little free ${{{{{{\rm{H}}}}}}}^{+}$ available as a proton source^13,30–34^. Markovic et al. proposed that alkaline HER kinetics may be tailored by tuning both HBE as well as the energy required to dissociate water, with the following RDS^30^:

6 $${{H}_{2}O+{e}^{-}\to H}^{*}+O{H}^{-}$$

In this picture, tuning surface oxophillicity promotes the dissociation of water at the surface by stabilization of ${{{{{\rm{O}}}}}}{{{{{{\rm{H}}}}}}}^{*}$, and oxophillic surface species such as ${{{{{\rm{Ni}}}}}}{\left({{{{{\rm{OH}}}}}}\right)}_{2}$ are proposed to accelerate water splitting on catalysts which have favorable HBE. By studying decorated step sites on Pt, McCrum et al. clarified how hydroxide binding energy (OHBE) affects the activation energy in the HOR/HER bifunctional mechanism^34^. On the other hand, Ledezma-Yanez and Sarabia et al. argue that alkaline HER may be limited by ${{{{{\rm{O}}}}}}{{{{{{\rm{H}}}}}}}^{-}$ transport through the double layer, where solvent structure has been thought to be more rigid and difficult to reorganize due to the interfacial electric field^11,17^. These authors attributed faster HER for Ni(OH)_2_ on Pt (111) to a decrease in interfacial field strength accompanying negative shifts in the PZC. Furthermore, Rebollar et al. showed that adding caffeine to Pt surfaces similarly shifts the PZC negatively and improves alkaline HOR and HER kinetics^35^. Rebollar et al. also observed a weakening of OHBE on Pt(pc) despite faster kinetics in the presence of caffeine, inconsistent with the OHBE theory. Although OHBE may be an important catalytic descriptor, it is not correlated with the reaction kinetics in every situation, and it appears that the electrode electrostatic potential is yet another kinetic descriptor.

Here, we showed that interfacial fields near the negatively charged Pt electrode do not appreciably impact solvent reorganization energy. Moreover, we show that solvation relaxation is slightly faster near the surface when it is negatively charged, indicating the solvent network does not become more rigid. Interestingly, our findings are consistent with experimental data of Rebollar et al.^35^. Using the HER/HOR kinetic isotope effect as a probe, the authors concluded that stronger interfacial fields near a negatively charged electrode did not cause sluggish solvent dynamics, rather, stronger interfacial fields were associated with slightly faster dynamics. Rebollar et al. then concluded that sluggish alkaline kinetics occur due to changes in the activation energy, not the Arrhenius prefactor. Our work and Rebollar’s findings together show that interfacial electric fields caused by the negatively charged electrode do not cause increased solvent redox reorganization energy or slow solvent dynamics.

We suggest that the modulation of ${{{{{{\rm{H}}}}}}}^{+}/{{{{{\rm{O}}}}}}{{{{{{\rm{H}}}}}}}^{-}$ interfacial electrochemical potentials by the charged surface directly affects redox activation barriers, which could explain why the PZC is correlated with HOR/HER kinetics. This argument is experimentally supported by recent estimates of electrostatic potential near the interface. Using a pH-sensitive probe reaction, Ryu and Surendranath showed that there is incomplete screening near the Outer Helmholtz Plane (OHP) on Pt/C at HOR/HER potentials up to a significant ionic strength of 280 mM^36^. The Volmer HOR/HER transition state is even closer to the surface, around $2-2.5 \, {{{{{\text{\AA }}}}}}$. At these small distances, the local electrostatic potential at the transition state is even more incompletely screened relative to the OHP and thus is strongly sensitive to the local electrostatic potential set up by the electrode. In our view, the change in the local electrostatic potential of H^+^, not changes in solvent polarization induced by the interfacial electric field, is a relevant factor that modifies the HOR/HER activation barrier.

We can outline a plausible mechanism by which the charged surface affects both alkaline HOR and HER kinetics. In base, since the Volmer equilibrium potential is much more negative than in acid, the reduced state is more favored relative to bulk ${{{{{{\rm{H}}}}}}}_{3}{{{{{{\rm{O}}}}}}}^{+}$, and ${{{{{\rm{O}}}}}}{{{{{{\rm{H}}}}}}}^{-}$—not ${{{{{{\rm{H}}}}}}}_{2}{{{{{\rm{O}}}}}}$—must directly aid the removal of ${{{{{{\rm{H}}}}}}}^{*}$ for HOR. When the electrode potential is below the PZC, just as we showed ${{{{{{\rm{H}}}}}}}^{+}$ is more energetically favorable near the surface, ${{{{{\rm{O}}}}}}{{{{{{\rm{H}}}}}}}^{-}$ will be less favorable near the surface. As Ramaswamy et al. earlier concluded, the energy penalty to bring ${{{{{\rm{O}}}}}}{{{{{{\rm{H}}}}}}}^{-}$ to the negative surface should result in higher HOR activation energy^14^. The HER activation energy is also expected to be larger when ${{{{{\rm{O}}}}}}{{{{{{\rm{H}}}}}}}^{-}$ is a product, since it is unfavorable for ${{{{{\rm{O}}}}}}{{{{{{\rm{H}}}}}}}^{-}$ to form near the negative surface as water splits. We note that in this work, we have not simulated concentrated solutions and therefore neglect the accumulation/depletion of H^+^ and OH^-^ in the double layer and their associated first-order kinetic rate dependence. Clearly, understanding the role of interfacial electrostatic potential in alkaline HOR/HER deserves further study. Studying how the ${{{{{\rm{O}}}}}}{{{{{{\rm{H}}}}}}}^{-}$ interfacial concentration responds to changes in local electrostatic potential and the interrelationship with supporting electrolyte effects will be left to future work.

Using controlled-potential Anderson-Newns Hamiltonian molecular dynamics, we have computed the diabatic and adiabatic free energies for the Volmer step on Pt (111). The Volmer step does not obey the linear-response approximation with respect to solvent polarization over the whole studied range, and polarization fluctuations around H* are much broader compared to ${{{{{{\rm{H}}}}}}}^{+}$. At physically relevant applied potentials below the PZC, solvent reorganization energy for the Volmer step is insensitive to interfacial electric fields, and solvation dynamics do not become more sluggish. As the surface becomes more negatively charged, we predict an increase in HBE due to electrostatic interactions between H* and the surface. Additionally, we find that the proton is highly attracted to the negative surface and cannot easily leave to bulk. In the reduction direction, stabilization of ${{{{{{\rm{H}}}}}}}^{+}$ by the negatively charged surface results in a late transition state where the solvent is farther from equilibrium ${{{{{{\rm{H}}}}}}}^{+}$ solvation polarization, resulting in a higher activation energy.

Since the relationship between the PZC and HOR/HER kinetics cannot be explained by changes in solvent reorganization energy or dynamics, we suggest that changes in the electrode electrostatic potential directly affect kinetics by changing ${{{{{{\rm{H}}}}}}}^{+}/{{{{{\rm{O}}}}}}{{{{{{\rm{H}}}}}}}^{-}$ electrochemical potentials in the double layer due to incomplete screening by the solution. Our findings help to clarify the role of interfacial electrostatic effects in hydrogen electrocatalysis and highlight the need to engineer the double layer and catalyst PZC for active alkaline catalysts.

## Methods

Here, we simulate the Volmer step on the Pt (111) surface to gain insight into interfacial-field dependent HOR/HER kinetics:

$${H}^{*}\leftrightarrow {H}^{+}+{e}^{-}$$

In heterogeneous electron transfer, the redox ion electronic state couples to the catalyst continuum of electronic states, and the ion orbital’s energy is additionally coupled to the fluctuating solvent. We treat this interaction approximately by utilizing solutions to the Anderson-Newns Hamiltonian, which has been successfully used to study heterogeneous ET reactions. The electronic Hamiltonian term is embedded in the molecular dynamics Hamiltonian to describe the electronically adiabatic reaction:

$${{{{{\mathcal{H}}}}}}={{{{{{\mathcal{H}}}}}}}_{{sol}}+{{{{{{\mathcal{H}}}}}}}_{{el}}$$

Where ${{{{{{\mathcal{H}}}}}}}_{{sol}}$ represents the Hamiltonian for the electrolyte-electrolyte, electrolyte-catalyst, and all other interactions not involving the redox ion electronic state. The electronic Hamiltonian term simplifies to yield an analytic form when we consider a single redox ion state with broadband coupling to the metal states:

$${E}_{0}\left(\Delta E\right)=	\frac{1}{2}\Delta E+1/\pi \left({\epsilon }_{a}+\Delta E-{\epsilon }_{f}\right){{{{{\rm{ta}}}}}}{{{{{{\rm{n}}}}}}}^{-1}\left[\frac{{\epsilon }_{f}-\left({\epsilon }_{a}+\Delta E\right)}{\Delta }\right] \\ 	+\frac{\Delta }{2\pi }{{{{{\rm{ln}}}}}}\left[{\left({\epsilon }_{a}+\Delta E-{\epsilon }_{f}\right)}^{2}+{\Delta }^{2}\right]$$

Where ${{{{{{\rm{\epsilon }}}}}}}_{{{{{{\rm{f}}}}}}}$ is the Fermi level of the metal, ${{{{{{\rm{\epsilon }}}}}}}_{{{{{{\rm{a}}}}}}}$ is the vacuum energy level of the hydrogen 1s state, Δ is the metal-ion coupling matrix element, and Δ*E* is the random variable for the vertical energy gap coordinate which tracks the collective solvent fluctuations. As the MD simulation proceeds, Δ and Δ*E* are calculated at every timestep and used to update the electronic term E_0_(Δ*E*) and the associated electronic forces. The form of *E*_0_ used here follows from the behavior of the expected occupancy of the hydrogen orbital state $({{\langle }}{n}_{a}\rangle )$, which varies with Δ*E* as follows:

$$\left\langle {n}_{a}\left(\Delta E\right)\right\rangle=\frac{1}{2}+\frac{1}{\pi }{{{\tan }}}^{-1}\left(\frac{{\epsilon }_{f}-\left({\epsilon }_{a}+\Delta E\right)}{\Delta }\right)$$

The expected occupancy smoothly switches from 0 (H^+^) to 1 (reduced H) as $\Delta {{{{{\rm{\it{E}}}}}}}$ decreases, i.e., accompanying proton desolvation. The charge transfer occurs most rapidly as $\Delta {{{{{\rm{\it{E}}}}}}}$ crosses the Fermi level, and the rate of change is proportional to the broadening, $\Delta$. We use the approximation of broadband coupling for $\Delta$ and fit the electronic coupling to an exponential function of distance from the electrode:

$$\Delta \left(z\right)={\Delta }_{0}{{\exp }}(-\beta z)$$

Where ${\Delta }_{0}=16\,{{{{{\rm{eV}}}}}}$ and $\beta=1.1{\;{{{{{\text{\AA }}}}}}}^{-1}$. These values are approximated using DFT calculations and provide a reasonable estimate of the broadening– 3.0 eV for adsorbed hydrogen. More detail about the derivation of the electronic term can be found in previous works by Grimley and Newns and in Supplementary Note 1^21,37,38^.

Briefly, we can summarize how hydrogen behaves in the adiabatic ET simulations with this embedded-ANH approach. In the adiabatic simulations, the H orbital occupancy is a continuous function of the electronic coupling, $\Delta$, and the fluctuating vertical energy gap, $\Delta E$. In the limit of $\left\langle {n}_{a}\right\rangle \to 0,$ the interaction potentials between the redox hydrogen and the solvent are that of H^+^. In the limit of $\left\langle {n}_{a}\right\rangle \to 1$, the H/solvent interaction potentials are that of reduced hydrogen. To bind H* to the surface, we add an additional Morse bond term between reduced H and the Pt surface. As hydrogen proceeds through the adiabatic Volmer transition state and is reduced near the surface, H forms a bond with Pt. Details about the H^+^ and H interaction potentials are available in Supplementary Note 1.

In the Volmer step, we model the reaction as proceeding along the relevant reaction coordinates of the collective solvent polarization, $\Delta E,$ and the hydrogen’s distance from the surface, $z$. We perform importance sampling along $\Delta E$ and *z* in canonical MD simulation at 300 K. All molecular dynamics simulations are performed in LAMMPS^39^. We simulate both the diabatic curves along $\Delta E$ at fixed *z* as well as the 2D adiabatic surfaces. In diabatic simulations, ${{{{{{\mathcal{H}}}}}}}_{{{{{{\rm{el}}}}}}}$ is not included in the total Hamiltonian, and the ion is fixed in one of the two states. For each diabatic curve, we used 33 umbrella sampling windows of length 60 ps each. Simulations for each window were performed in series, with each simulation picking up from the last snapshot of the prior adjacent window. Equilibration for the initialized system lasted 2 ns and subsequent equilibration for the first bias window lasted 60ps. The restraining potential was smoothly ramped from one window to another initially and subsequent equilibration lasted 6 ps. Unbiased free energies were estimated using the standard weighted histogram analysis method^40^. For 1D diabatic curves, block averaging of the window means was performed to estimate error and ensure adequate sampling. The standard error in the diabatic reduction free energy change $\Delta F={F}^{{{{{{\rm{red}}}}}}}\left(\Delta {e}_{{{\min }}}^{{{{{{\rm{red}}}}}}}\right)-{F}^{{{{{{\rm{ox}}}}}}}(\Delta {e}_{{{\min }}}^{{{{{{\rm{ox}}}}}}})$ for redox at fixed z is estimated to be ±0.03 eV using the procedure from Zhu et al.^41^. For the 2D adiabatic surfaces, similar harmonic restraint window settings were used to sample along $\Delta E$, and we additionally sample along the z coordinate in window increments of 0.2 Å. Errors in calculated adsorption energies are similarly estimated to be $\pm 0.03{{{{{\rm{eV}}}}}}$.

The interaction potentials for the ${{{{{{\mathcal{H}}}}}}}_{{{{{{\rm{sol}}}}}}}$ terms include both the solvent-solvent and solvent-metal contributions. For the solvent-solvent contribution, we use TIP3P waters^42^. For the water-metal potential, the noncoulombic interactions are modeled using the Siepmann and Sprik force field^43^, and the coulombic interactions are treated using the constant potential method from Voth’s group^22^. Details of the constant potential method are provided in the supplementary methods. For all short-range interactions, we use a cutoff of 12.0 Å. Long-range coulomb interactions are computed using the PPPM solver with an RMSE force accuracy setting of $0.14{{{{{\rm{meV}}}}}}/\;{{{{{\text{\AA }}}}}}$ in LAMMPS. The k-space slab correction is used for long-range electrostatics in the z direction with a slab vacuum length of 5.0x the cell z length. A small timestep of 0.3 fs was used due to the fast motion of the proton and provided good energy conservation.

For the capacitor cell geometry, the electrolyte region is defined by a box of size ${{{{{\rm{x}}}}}}=24.0\;{{{{{\text{\AA }}}}}},\,{{{{{\rm{y}}}}}}=24.9\;{{{{{\text{\AA }}}}}},\,{{{{{\rm{z}}}}}}=50.0\;{{{{{\text{\AA }}}}}}$. Two Pt electrodes with exposed (111) facets are placed on either side of the electrolyte, each containing 3 layers of Pt atoms. The electrolyte contains 960 rigid waters and yields an average density equal to the bulk density of TIP3P waters, ${{{{{\rm{\rho }}}}}}=0.98\,{g\; c}{m}^{-3}$. The electrolyte Pt atoms are frozen and not integrated in the Verlet algorithm. The top layer of Pt atoms on each electrode is chosen as the conducting plane where constant potential is maintained.

## Supplementary information

Supplementary Information

## Data Availability

The authors declare that the datasets generated during the current study are included in this published article and its supplementary information files. While molecular dynamics trajectory data are not included with the published article due to their large disk size, they are available from the corresponding author upon reasonable request. Source data are provided with this paper.

## Source data

Source Data

## Code availability

Computer code files written for this study are not published online due to use of code dependencies that are not publicly available, however, the code can be provided by the corresponding author upon reasonable request.
