# Supplementary material for: Insights into solvent and surface charge effects on Volmer step kinetics on Pt (111)
Source: Nat Commun. 2023 Apr 25;14:2384. doi: 10.1038/s41467-023-37935-6 (PMC10130056; doi:10.1038/s41467-023-37935-6)
Supplement: Supplementary file 1 — Supplementary Information [file 41467_2023_37935_MOESM1_ESM.pdf]

Supplementary information: Insights into Solvent and Surface Charge Effects on Volmer Step Kinetics on Pt (111)

Jon C. Wilson[1,2], Stavros Caratzoulas[1,2], Dionisios G. Vlachos[1,2], Yushan Yan[1]

1) Department of Chemical and Biological Engineering, University of Delaware, 150 Academy St, Newark, DE 19713 USA,
2) Catalysis Center for Energy Innovation, University of Delaware, 221 Academy St, Newark, DE 19716, USA.

Corresponding Authors: yanys@udel.edu, vlachos@udel.edu, cstavros@udel.edu

Supplementary Note 1.

Theory. To motivate our choice of model features, we briefly recap key theory developments leading up to the current understanding of hydrogen electrocatalysis. R. A. Marcus formulated a general theory of electron transfer which now has much well-established experimental support and broad applicability to a wide range of electron transfer (ET) systems. In Marcus theory, redox systems display parabolic free energy curves with respect to the solvent polarization, and charge transfer is most likely near the crossing point of the two redox states for non-adiabatic ET in the weak coupling limit[1].

In heterogenous ET systems, there is frequently strong ion-metal coupling resulting in adiabatic ET. For hydrogen chemisorption which involves strong ion-metal coupling, the Anderson-Newns Hamiltonian (ANH) approach clarified the relationship between the electronic structure of catalysts and the physics of chemisorption[2]. Further, Santos and Schmickler developed an ANH-based model which incorporated linear ion-solvent coupling as well as ion-metal electronic coupling for adiabatic ET. The Anderson-Newns-Schmickler model clarified how the electronic structure of transition metals affects the free energy surface in hydrogen electrocatalysis when solvent reorganization is included[3]. Voth et al. developed an embedded ANH approach, embedding a weak electronic coupling approximate solution to the ANH into classical molecular dynamics, thereby incorporating an atomistic simulation of electrochemical double layer (EDL) effects into adiabatic ET studies[4]. Recently, Lam and the Hammes-Schiffer group formulated an empirical valence bond theory model to study Volmer proton coupled electron transfer in the electronically adiabatic regime[5]. In the Lam et al. model, solvent interactions are treated under the linear-response approximation, and additionally, an electrode potential and a mean-field electrostatic model of the EDL are included[5]. The inclusion of the interfacial electric field has a significant effect on Volmer free energies. For example, it was shown that after PCET, the conjugate base of the proton donor moves away from the surface due repulsion from a negatively charged electrode. Modeling ion-solvent electronic coupling, solvent reorganization, and electrode potential together is a grand challenge in electrochemistry theory. Theoretical efforts to incorporate all these essential features in a comprehensive electrocatalysis model are ongoing[6]. These significant works are still only a small sampling of a wide body of theoretical efforts which have been reviewed in more detail elsewhere[6].

One important feature of the hydrogen electrocatalysis models by Schmickler et al. and Hammes-Schiffer et al. is that the effects of surface electrostatic potential are either not included explicitly or treated using mean-field approximations[3,5]. In this work, we take a different approach and extend Voth's embedded ANH model to simulate the Volmer step, treating the solvent degrees of freedom classically with molecular dynamics[4]. We additionally incorporate conducting interface boundary conditions which enable control over the cell voltage in a capacitor cell configuration, and we define the vertical excitation coordinate appropriately in the presence of polarized metal atoms[7]. This approach is complementary to other works which treat the strong metal-ion electronic coupling in more detail[5,8]. Here, we treat metal-ion coupling in a simplified way, choosing instead to focus on understanding interfacial electrostatics and double layer effects, which are less well understood in the context of the Volmer step.

Electronic Hamiltonian details. The electronic Hamiltonian term is added to the molecular dynamics Hamiltonian to describe the electronically adiabatic charge transfer:

$$\mathcal{H} = \mathcal{H}_{sol} + \mathcal{H}_{el}$$

Initially, the electronic Hamiltonian term $\mathcal{H}_{el}$ can be written in the second quantized formalism as follows:

$$\mathcal{H}_{el} = (\epsilon_a + \Delta E)n_a + \sum_k (\epsilon_k n_k + \mathcal{V}_{ak} c_a^\dagger c_k + \mathcal{V}_{ka}\, c_k^\dagger c_a)$$

Where ϵ_a is the vacuum energy level of the hydrogen orbital $|a\rangle$. We assume there is a single H orbital state that interacts with the continuum of states on the metal. This single hydrogen orbital has occupancy n_a which has values 0 or 1. ΔE is the vertical energy gap collective coordinate. The summation over the k electronic states includes contributions from occupied metal states as well as ion-metal interaction terms representing the charge transfer between the ion orbital to the metal states. V_{ak} are the electronic resonance integrals between $|a\rangle$ and $|k\rangle$, and $c_a^\dagger, c_a, c_k^\dagger, c_k$ are the electron creation and annihiliation operators for the respective states.

To embed the electronic term into molecular dynamics, an analytic form for $\mathcal{H}_{el}$ is needed. Thus, we must make simplifying assumptions about the electronic interaction between the hydrogen state and the metal states. We make the adiabatic assumption of separability between the solvent and electronic degrees of freedom. It has been shown that the electronic Hamiltonian can be simplified to yield the following analytic form when we consider a single redox ion state with broadband coupling to the metal:

$$E_0(\Delta E) = \frac{1}{2}\Delta E + \frac{1}{\pi}(\epsilon_a + \Delta E - \epsilon_f)\tan^{-1}\left[\frac{\epsilon_f - (\epsilon_a + \Delta E)}{\Delta}\right] + \frac{\Delta}{2\pi}\ln\left[(\epsilon_a + \Delta E - \epsilon_f)^2 + \Delta^2\right]$$

Where ϵ_f is the Fermi level of the metal and Δ is the coupling matrix element which lowers the transition state energy and enables electrocatalysis. E_0 is computed at each MD timestep for the adiabatic electron transfer calculations. The level-broadening parameter Δ is the coupling between the hydrogen state and the k metal states and is formally given by:

$$\Delta(\epsilon) = \pi \sum_k |V_{ak}|^2 \delta(\epsilon - \epsilon_k)$$

With the broadband coupling assumption, Δ becomes independent of energy. Since the magnitude of electronic coupling increases as the hydrogen atom moves toward the surface, we make the first order approximation of exponential decay with respect to z as noted in the main text.

Constant potential method. To model the electrified interface, we incorporate a method of enforcing controllable constant-potential boundary conditions in molecular dynamics. We adopt Voth's protocol which utilizes two sets of explicit primary image charges in a capacitor-cell configuration[7]. The conducting image planes are chosen as the center of the exposed layer of metal atoms on each electrode. The nonuniform portion of surface charge distribution is modeled by the primary image charges. The excess free charge Q_0 necessary to set a voltage ΔV_0 in a cell containing electrolyte is the same as for an equivalent empty capacitor:

$$\Delta V_0 = \frac{Q_0}{\epsilon_a A} D$$

Where D is the cell gap width and A is the surface area. The higher-order images and excess charge are distributed evenly across the surface through an efficient method as follows:

$$q_{LE} = \sum_{i=1}^{n} \frac{q_{R_i} z_{R_i}}{D} + Q_0$$

$$q_{RE} = \sum_{i=1}^{n} q_{R_i} \left(1 - \frac{z_{R_i}}{D}\right) - Q_0$$

Where q_{R_i} and z_{R_i} are the real electrolyte charges and z positions in the cell, respectively. q_{LE} is the charge on the left electrode and q_{RE} is for the right electrode. At each timestep, the primary image charges and uniform charges are updated. As redox occurs in adiabatic ET, the ion's primary images as well as the uniform charges are similarly updated to reflect the changing charge state of the ion due to the ANH, $q_H = 1 - \langle n_a \rangle$.

Vertical energy gap and interaction potentials. Next, we define the collective coordinate for solvent reorganization. As previous work has shown, it is convenient to adopt the diabatic vertical energy gap ΔE between the reduced and oxidized diabatic states as the collective coordinate. The vertical energy gap is the potential energy required to change a particle from one redox state to another, keeping all nuclear coordinates **R** fixed.

First, it is instructive to write out the interaction potential terms which describe the interaction between the ion and its images with the rest of the system. The Coulombic terms are as follows:

$$\mathcal{V}_{\text{coulombic}} = (z - n_a)\big(\mathcal{V}_{\text{elyte−ion}} - \mathcal{V}_{\text{elyte−ion images}} - \mathcal{V}_{\text{elyte images−ion}} + (z - n_a)\mathcal{V}_{\text{ion−ion images}} + \mathcal{V}_{\sigma\text{−ion}}\big) + \mathcal{V}_{\sigma\text{−water}}$$

Where $\mathcal{V}_{\text{elyte−ion}}$ is the Coulomb interaction between the ion and the rest of the electrolyte (excluding the ion itself), $\mathcal{V}_{\text{elyte−ion image}}$ is between the electrolyte and the ion images, $\mathcal{V}_{\text{elyte images−ion}}$ is between the electrolyte images and the ion, $\mathcal{V}_{\text{ion−ion imgs}}$ is between the ion and its own images, $\mathcal{V}_{\sigma\text{−ion}}$ is between the ion and the uniform electrode charges q_{LE} and q_{RE}, and $\mathcal{V}_{\sigma\text{−water}}$ is between the electrolyte and the uniform electrode charges. z is the charge of the ion, +1 for the proton.

When defining ΔE, it is often assumed that nonpolar interaction potentials are the same in the reduced and oxidized state of an ion. However, hydrogen is a special case because the solvated proton coordinates strongly with water, whereas reduced H-water interactions are repulsive. Therefore, we have fit simple functional forms to approximate the non-Coulombic contribution to hydrogen-water interactions for the reduced and oxidized states. We add additional terms to the overall potential for the two redox states:

$$\mathcal{V}_{\text{elyte−ion np}} = n_a \mathcal{V}^{H}_{\text{elyte−ion np}} + (1 - n_a)\mathcal{V}^{H^+}_{\text{elyte−ion np}}$$

$\mathcal{V}^{H}_{\text{elyte−ion np}}$ are the nonpolar interactions between reduced H and the electrolyte, and $\mathcal{V}^{H^+}_{\text{elyte−ion np}}$ are the nonpolar interactions between the proton and the electrolyte.

$$\mathcal{V}^{H}_{\text{elyte−ion np}} = \sum_{i}^{n_o} A^{H}_{O} \exp(-B_{OH} r_i) + \sum_{j}^{n_H} A^{\text{H}}_{H} \exp(-B_{HH} r_j)$$

$$\mathcal{V}_{\text{elyte-ion np}}^{H^+} = \sum_{i}^{n_o} \left(D_O^{H^+} \left(1 - \exp\left(-\alpha_O^{H^+} (r_i - r_0)\right)\right)^2 - D_O^{H^+} \right) + \sum_{j}^{n_H} A_H^{\mathrm{H}^+} \exp\left(-B_H^{H^+} r_j\right)$$

Where i runs over all water oxygen atoms and j over all water hydrogens. We have parametrized the interaction potentials based on Gaussian calculations of small clusters of water solvating a single proton at the B3LYP/aug-cc-pvdz theory level. With our potential, solvating the proton in a cluster of two waters produces a Zundel-like ion with oxygen-proton bond length of 1.22 Å, matching well with the ab-initio calculated distance[9]. The solvation enthalpy of the proton in a cluster of 128 TIP3P waters was calculated to be -10.7eV using our force field, reasonably close to ab-initio estimates for the bulk solvation enthalpy of -11.4eV to -11.9eV[9].

Additionally, there is a chemical bond contribution between hydrogen and the catalyst surface. Assuming a broadband coupling in our model does not sufficiently capture the chemisorption of H in the model; the Hilbert transform of Δ gives the level shift of the hydrogen DOS, Λ, sometimes called the chemisorption function. Λ becomes zero when Δ is constant, and consequently hydrogen is not stabilized as it approaches the surface. To correct for the simplified coupling and make reduced H adsorb on the surface, we have added an additional platinum-hydrogen Morse bond term, fitted from DFT calculations in VASP. In the DFT calculations, the energies of hydrogen were calculated at several distances above a Pt (111) atop site for a relaxed 3x3x4 slab. We used the PBE functional, 7x7x1 Γ-centered kpoint mesh, 1st order Methfessel-Paxton smearing with width 0.15 eV, and a 500 eV planewave cutoff. We assume that the platinum-H interactions in the reduced state can be described by a z-dependent bonding term. By multiplying the potential with the orbital occupancy n_a, we create a switching function that describes the bond-breaking and formation as the oxidation state changes:

$$\mathcal{V}_{\text{Pt-H}} = n_a \left(D_{\text{Pt}}^{\text{H}} \left(1 - \exp\left(-\alpha_{\text{Pt}}^{\text{H}} (z - z_0)\right)\right)^2 - D_{\text{Pt}}^{\text{H}} \right)$$

where D_{Pt}^{H} is the equilibrium bond energy, $\alpha_{\text{Pt}}^{\text{H}}$ is the decay constant, and z_0 is the equilibrium position for H on the Pt surface. This bond-breaking switching approximation has been used in the past to describe dissociative adsorption in similar MD models by Calhoun[10]. In the reduced state, $\mathcal{V}_{\text{Pt-H}}$ takes a Morse potential form, and it is zero in the oxidized state; instead, the attraction between the proton and the surface takes the form of the well-known image potential, which is grouped with the Coulombic terms. Interaction potential terms not relevant to the electronic Hamiltonian are grouped in with $\mathcal{H}_{\text{sol}}$ because these $\mathcal{H}_{\text{sol}}$ terms cancel out in the vertical energy gap for the system. Now, we have the total potential for the hydrogen ion:

$$\mathcal{V} = \mathcal{V}_{\text{coulombic}} + \mathcal{V}_{\text{Pt-H}} + \mathcal{V}_{\text{elyte-ion np}}$$

ΔE is defined as the difference between the reduced and oxidized states, i.e., occupancy 1 and 0:

$$\begin{aligned}\Delta E(\mathbf{R}) &= \mathcal{V}^{\text{red}}(\mathbf{R}) - \mathcal{V}^{\text{ox}}(\mathbf{R}) \\ &= -\left(\mathcal{V}_{\text{elyte-ion}} - \mathcal{V}_{\text{elyte-ion images}} - \mathcal{V}_{\text{elyte images-ion}} + (2z-1)\mathcal{V}_{\text{ion-ion imgs}}\right) \\ &\quad + (z-1)\mathcal{V}_{\sigma\text{-ion}}^{\text{H}} - (z)\mathcal{V}_{\sigma\text{-ion}}^{\text{H}^+} + \left(\mathcal{V}_{\text{elyte-ion np}}^{\text{H}} - \mathcal{V}_{\text{elyte-ion np}}^{H^+}\right) + \mathcal{V}_{\text{Pt-H}}\end{aligned}$$

where $\mathcal{V}_{\sigma\text{-ion}}^{H}$ represents the $\mathcal{V}_{\sigma\text{-ion}}$ term when the electrode is polarized in accordance with the reduced state charge, and $\mathcal{V}_{\sigma\text{-ion}}^{H^+}$ is similarly defined for the oxidized state charge. We exclude the contribution to ΔE from $\mathcal{V}_{\sigma\text{-water}}$, since it is an unphysical contribution from the higher-order image charges that arise from the microscopic capacitor configuration. In a real macroscopic electrochemical cell, there are no

higher order images to be adjusted as redox occurs, thus there is no higher-order image contribution to the fluctuating ion orbital energy. However, in the simulation, the surface charges q_{LE} and q_{RE} are correctly updated as q_H varies for the purpose of maintaining the correct potential difference ΔV. A summary of the interaction parameters used is shown in supplementary table 1:

Supplementary Table 1. Interaction parameters for the redox ion-system potentials.

Parameter	Value
A_O^H	1.10 eV
B_O^H	1.15 Å^{-1}
A_H^{H}	3.50 eV
B_H^H	2.60 Å^{-1}
$D_O^{H^+}$	1.50 eV
$\alpha_O^{H^+}$	2.50 Å^{-1}
r_0	1.24 Å
$A_H^{\mathrm{H}^+}$	50.0 eV
$B_H^{H^+}$	12.5 Å^{-1}
D_{Pt}^H	5.81 eV
α_{Pt}^H	1.00 Å^{-1}
z_0	0.71 Å

Supplementary Note 2.

Electrode potentials and surface charging relation. Here we explain the relationship between the cell potential and the single-electrode potentials and their surface charge. During the simulation, the induced surface charges on the two electrodes fluctuate to maintain constant potential as the electrolyte species move. The constant potential algorithm allows us to control the potential difference ΔV across the capacitor cell with spacing D while maintaining overall charge neutrality for the system. If we consider an equivalent empty capacitor with cell potential ΔV, the cell is symmetric with field $\Delta V/D$, so we can define a vacuum reference as 0 V in the center of the cell which does not vary with ΔV. Because of the symmetry, the electrode electrostatic potentials can be defined as equal and opposite on an absolute scale versus the reference:

$$V_- = -\frac{\Delta V}{2}$$

$$V_+ = \frac{\Delta V}{2}$$

Where V_- and V_+ are the negative and positive electrode potentials. When the empty capacitor is uncharged, both electrode potentials are equal to the reference, that is, $V_- = V_+ = V_{\mathrm{pzc}} = 0$ V when $\Delta V = 0$ V. As defined, the potentials of the respective electrodes relative to the vacuum or pzc are unrelated to the presence of electrolyte, so the above absolute potential relations are well-defined in the electrolyte-filled cell.

The surface charging behavior of each electrode depends strongly on the electrolyte response. The average surface charge on each electrode is related to the double layer capacitance C_{dl}:

$$\langle\sigma\rangle(V) = \int_{V_{\mathrm{pzc}}}^{V} C_{dl}(v)dv$$

Where $\langle\sigma\rangle$ is the ensemble average of the surface charge density and V_{pzc} is the potential of zero free charge. In Figure S1, we show the capacitor configuration, spatial profile of electrostatic potential, and double layer charging relation for different voltages.

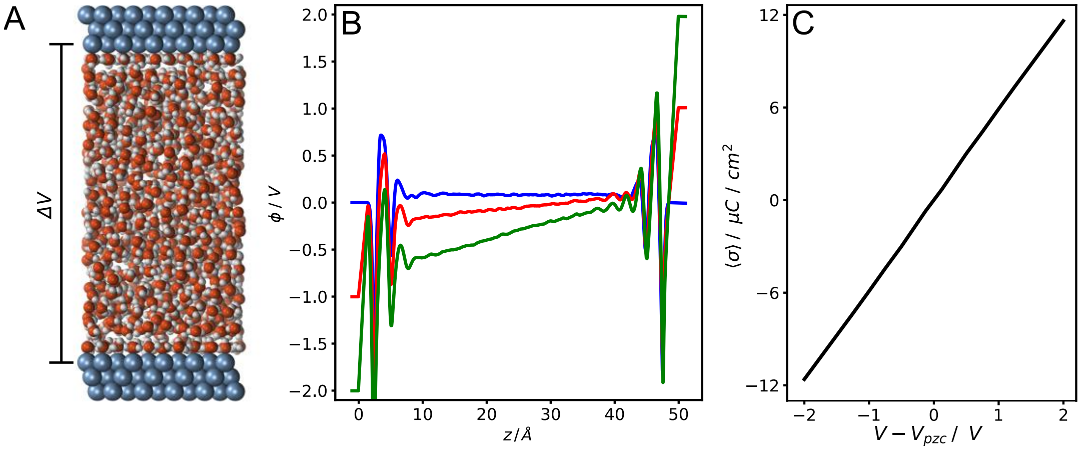

Figure S1. Potential profile and double charging relation in capacitor cell with neat water. A) Diagram of capacitor cell with gap $D = 50$ Å between image planes. Waters are placed between two slabs with Pt (111) facets exposed. B) Planar-averaged Poisson potential along the length of the capacitor at various applied voltages, C) Average surface charge as a function of applied potential, $V-V_{pzc}$. The slope of the line is the double layer capacitance, $C_{dl} \cong 6\ \mu F/cm^2$, and is due to reorientation of water molecules. (In B, the blue, red, and green curves correspond to ΔV=0 V,1 V, and 2 V respectively.)

Figure S1B shows the correct ΔV is established across the cell, with significant fluctuations in potential due to the orientation of water in the double layer. Moreover, the potential drop across the double layer is asymmetric with respect to the sign of voltage. Adsorbed water naturally tends to orient with its dipole pointing slightly away from the Pt electrodes, but in-plane hydrogen bonding aligns O-H bonds parallel to the surface. Thus, water dipoles more easily rotate away from the positive surface than towards the negative surface, hence the potential drop across the positive electrode double layer is larger.

In Figure S1C, we show the double layer charging behavior. C_{dl} is nearly constant for the potentials studied and lacks the bell-shaped curve observed on Pt, likely because this model does not reproduce the potential-induced desorption of water[11]. Nevertheless, the classical simulation with image charges maintains constant cell voltage as expected.

Supplementary Note 3.

Water equilibrium orientation around H⁺. In Figure S2 we plot the equilibrium orientation of water molecules around the H^+ at a fixed position, z, relative to the surface for various electrode potential values. The orientation of the water is defined by the angle

$$\cos(\theta) = \mathbf{r}_{\mathrm{H^+-O}} \cdot \boldsymbol{\mu}_{\mathrm{H_2O}}$$

where $\mathbf{r}_{\mathrm{H^+-O}}$ is the vector between the proton and a water oxygen in the proton's solvation shell, and $\boldsymbol{\mu}_{\mathrm{H_2O}}$ is the dipole vector of that solvating water. Only water molecules within 3.2 Å of the proton are included. In panels A, B and C, the proton is fixed at z = 6 Å and the electrode potential is 0, -1 and -2 V, respectively. Note how the three distributions are statistically similar, indicating that the electrode field has a minimal effect on the equilibrium polarization of the water solvating the proton. In S2D-F, the proton is fixed at z = 2 Å and the electrode potential is 0, -1 and -2 V, respectively. Even at such short distance from the surface, the equilibrium orientation polarization of the solvating water is essentially determined by the proton's strong field. The distributions P(θ) become noticeably more localized as the proton approaches the surface due to desolvation.

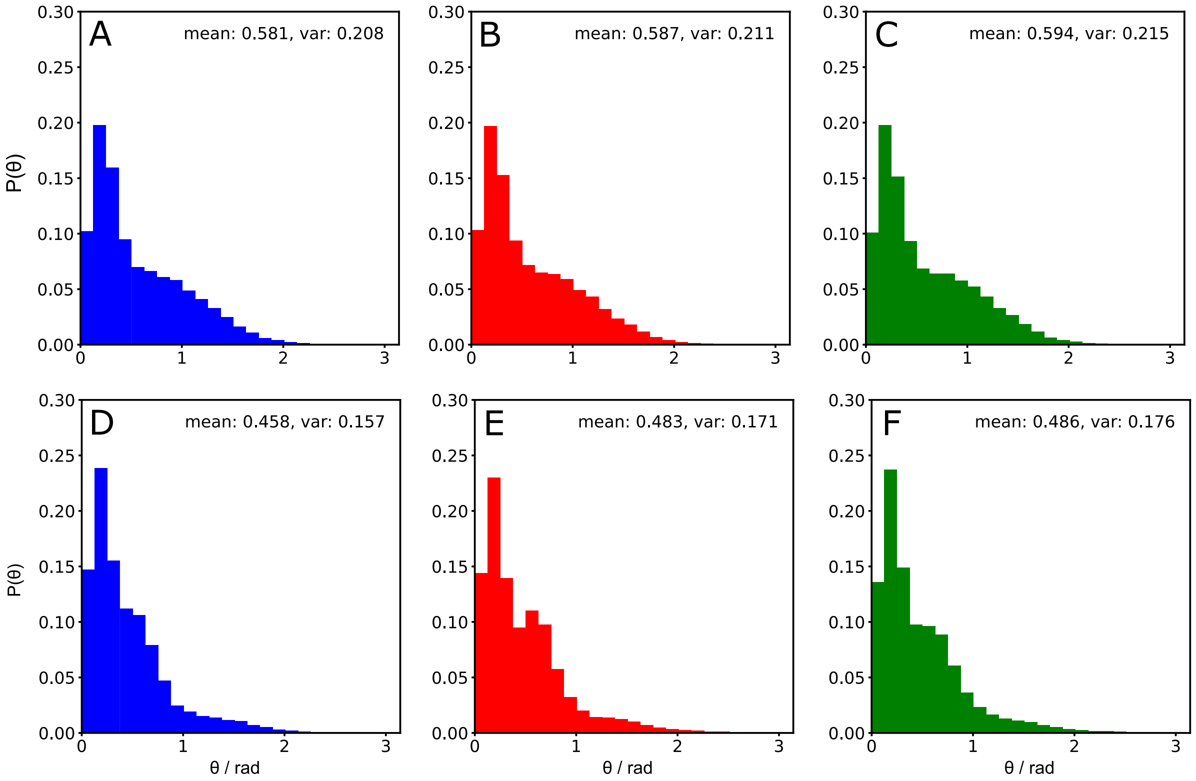

Figure S2. Angular distribution functions of waters solvating the proton. Statistics collected for the redox H oxidized diabatic state at fixed z. A), B), C): $z = 6.0$ Å and D), E), F): $z = 2.0$ Å for three different voltages. $V - V_{\mathrm{pzc}} = 0$ V, -1 V, -2 V correspond to blue, red, and green, respectively.

Supplementary Note 4. Potential-dependent interfacial water configurations. Since the structure of the interfacial solvent plays an important role in the electrostatics and solvation of ions in the double layer, it is important to accurately model Pt-water interactions. Here we have used the force field from Siepmann and Sprik, which has been widely used for the Pt (111) surface and produces realistic water configurations. Here we show how adsorbed waters respond to changes in the applied voltage. The trends in water reorientation and hydrogen bonding that we observe here are qualitatively similar to results by Li et al. for water on Au, obtained using a combination of AIMD and Raman spectroscopy[12].

In Figure S3A, snapshots of the surface and first layer of waters are shown at varying voltages. At $V - V_{\text{pzc}} = 0\ V$, water OH bonds primarily hydrogen bond in-plane with neighboring waters. A few OH bonds point up towards the solution, i.e. “H-up” waters. From the z-distributions of water hydrogens in S3C, the number of H atoms around z = 2.3 Å and 3.2 Å decreases at negative $V - V_{\text{pzc}}$, but increases around z = 1.5 Å. In S3D, the distribution of angles between the water dipoles and surface normal are shown. Values closer to 1 correspond to H-up waters. As expected, there is a shift from more H-up to more H-down waters at negative voltage.

In S3E we show the distribution of the number of hydrogen bonds (donors + acceptors) averaged over all surface waters. The criteria we use for a hydrogen bond between two water molecules is when the interoxygen distance between neighboring waters is less than 3.5 Å and the H−O···H angle is <30°. At the PZC, most surface waters have 3 or 4 hydrogen bonds. At negative electrode potentials, the number of waters with only 1 or 2 hydrogen bonds increases; there are fewer hydrogen bonds parallel to the surface and fewer hydrogen bonds between adsorbed waters and waters above the surface. The disruption of hydrogen bonding slightly lowers the coverage of water (S3B), and more waters occupying 3-fold surface sites are visible in S3A.

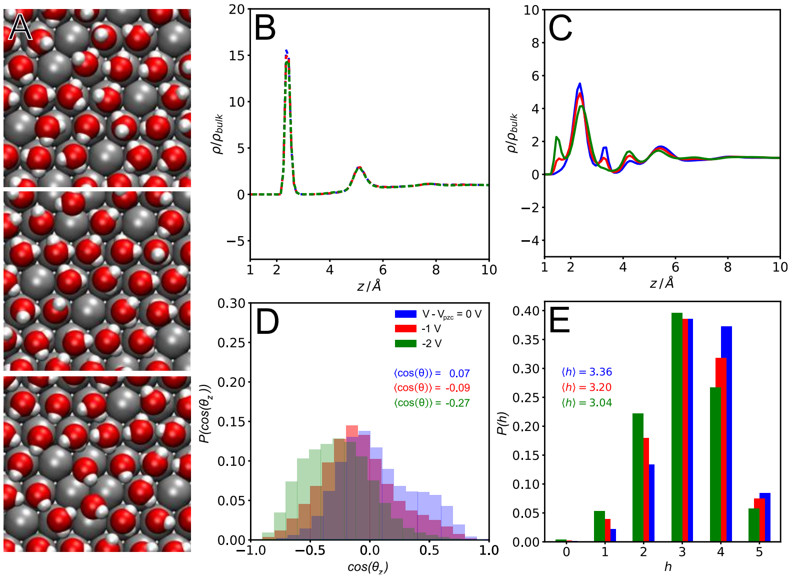

Figure S3. Potential-dependent interfacial water configurations. A) Characteristic snapshot of adsorbed water configurations at different voltages $V - V_{\text{pzc}} = 0\ \text{V}, -1\ \text{V}, -2\ \text{V}$ (top to bottom). B) z-distribution functions of oxygen atoms belonging to water. C) z-distribution functions of hydrogen atoms belonging to water. D) distribution of $\cos(\theta_z)$ of adsorbed water’s dipole angles with respect to the surface normal. E) probability distribution of the numbers of hydrogen bonds (donors + acceptors) per adsorbed water.

Supplementary Note 5.

Solvation water dipole autocorrelation relaxation. In Figure S4 we plot the dipole moment autocorrelation function $C_{\mu\mu}(t) = \langle \delta\boldsymbol{\mu}(t) \cdot \delta\boldsymbol{\mu}(0) \rangle / \langle \delta\boldsymbol{\mu}(0)^2 \rangle$ for the water molecules in the first solvation shell of the proton at two positions, z, relative to the electrode, and for three values of the electrode potential; $\delta\boldsymbol{\mu}(t)$ is the fluctuation from the mean. Only water molecules within 3.2 Å of the proton are included. Evidently, there is a distribution of solvation water relaxation times as a function of the distance from the surface. Far from the surface, *z* = 6 Å (Figure S4A), the collective nuclear motion of the solvating waters results in faster relaxation than at *z* = 3 Å (Figure S4B) where, because of the confinement of water and depletion of the proton solvation shell due to the proximity to the surface, there is insufficient number of water molecules to achieve cooperativity and fast relaxation. In the latter case, after the initial fast librational relaxation, the slow orientation relaxation is essentially determined by the slow response of a single molecule[13]. Furthermore, we see once again that the electrode field has an insignificant effect on the relaxation time, which is primarily determined by the strong field of the proton. Thus, neither the equilibrium distribution of water dipoles around the proton (see Supplementary Note 4) nor the dynamics of the solvation water are influenced by the electrode field and this is reflected in the essentially unchanging solvent reorganization energy with electrode potential.

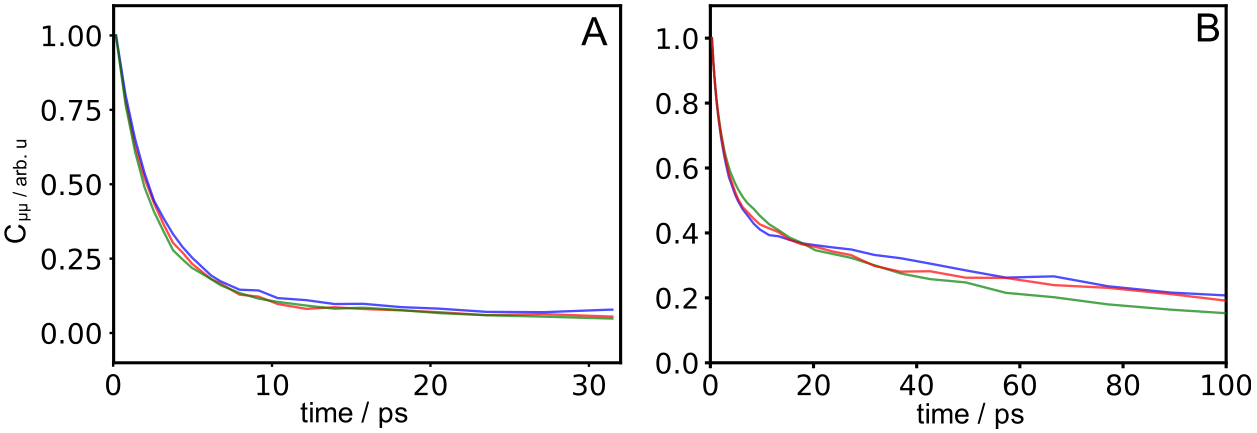

Figure S4. Dipole moment autocorrelation functions for waters solvating the proton. The results are plotted for the proton at fixed *z* where A) *z* = 6.0 Å B) *z* = 3.0 Å for three different voltages. $V - V_{\mathrm{pzc}} =$ 0 V, −1 V, −2 V shown in blue, red, and green, respectively.

Supplementary Note 6.

Proton electrostatic potential autocorrelation functions. Since ion-solvent coupling is strong along the Volmer reaction coordinate, it is important to understand solvent dynamics, which can affect the shape of the transition state and recrossing rates beyond the classical transition state theory approximation. In the present regime of strong solute-solvent coupling, the electron transfer rate constant is inversely proportional to the solvation time, i.e. $\tau^{-1} \propto k_{et}$ [14]. To estimate the collective solvation relaxation time, here we calculate the autocorrelation function for the proton's local electrostatic potential, where the proton is placed at fixed z above the electrode. The electrostatic potential autocorrelation function is calculated as follows[13]:

$$C_{\phi\phi} = \langle \delta\phi(t)\delta\phi(0) \rangle$$
$$\delta\phi = \phi(t) - \langle \phi \rangle$$

Where $\delta\phi$ is the instantaneous deviation from the ensemble average of the potential. We also estimate the mean relaxation time for this process by integrating the normalized autocorrelation function[13]:

$$\langle \tau \rangle = \int_0^\infty C_{\phi\phi}(t)dt$$

Where the integral is truncated to 3ps to avoid integrating through long times which have large sampling error. $\langle \tau \rangle$ is thus a lower bound estimate of the mean solvation time. Close to the surface, solvation dynamics are largely unaffected by the strong interfacial field (Figure S5A), likely because adsorbed waters are already highly confined by the surface. Further from the surface, the stronger interfacial field slightly accelerates solvation due to hydrogen bonding disruption, however, the effect is not particularly significant because the large proton electric field exerts a much greater influence on the dynamics of solvating waters (Figure S4B, S4C).

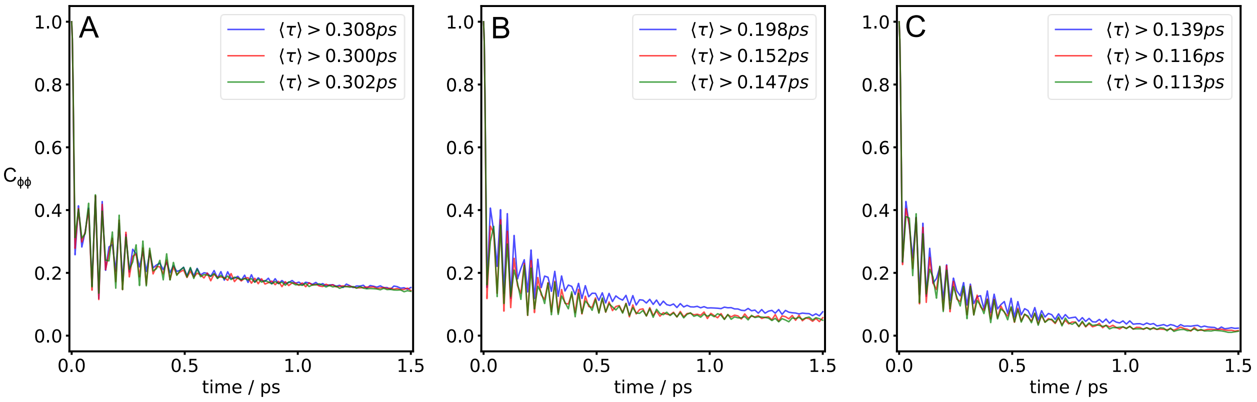

Figure S5. Autocorrelation for electrostatic potential of the proton at fixed z. The rate of relaxation of the electrostatic potential is proportional to the solvation time of the ion. Close to the surface, the solvation becomes more sluggish due to confinement of water on Pt (111). At more negative potentials, the slow water relaxation at long times is slightly faster just above the inner Helmholtz layer. A) $z = 2.0$ Å, B) $z = 4.0$ Å, C) $z = 6.0$ Å. $V - V_{\mathrm{pzc}} = 0$ V, -1 V, -2 V shown in blue, red, and green, respectively.

Supplementary Note 7.

Effect of overpotential on adiabatic ET. In Figure S6, we plot results from computing the Volmer adiabatic free energy surfaces where the electrode electrostatic potential $V - V_{\mathrm{pzc}}$ varies along with the overpotential, e.g. at $V - V_{pzc} = -1$ V, the reduction overpotential is 1eV larger than at $V - V_{\mathrm{pzc}} = 0$ V. Shifting the Fermi level higher biases the reaction to reduction (S6B), and the reduction transition state occurs earlier (S6A), indicating a shrinking reduction charge transfer coefficient. Additionally, the more negative electrostatic potential attracts H^+ to the surface. Interestingly, the reduction was expected to be barrierless at an overpotential of -1 V, however, we still observe a small barrier because the H^+ state near the surface is stabilized by the negatively charged surface. In essence, the decrease in the H^+ reduction activation barrier with increasing overpotential is less than expected due to the poorly screened negative surface charge, which favors the oxidized state.

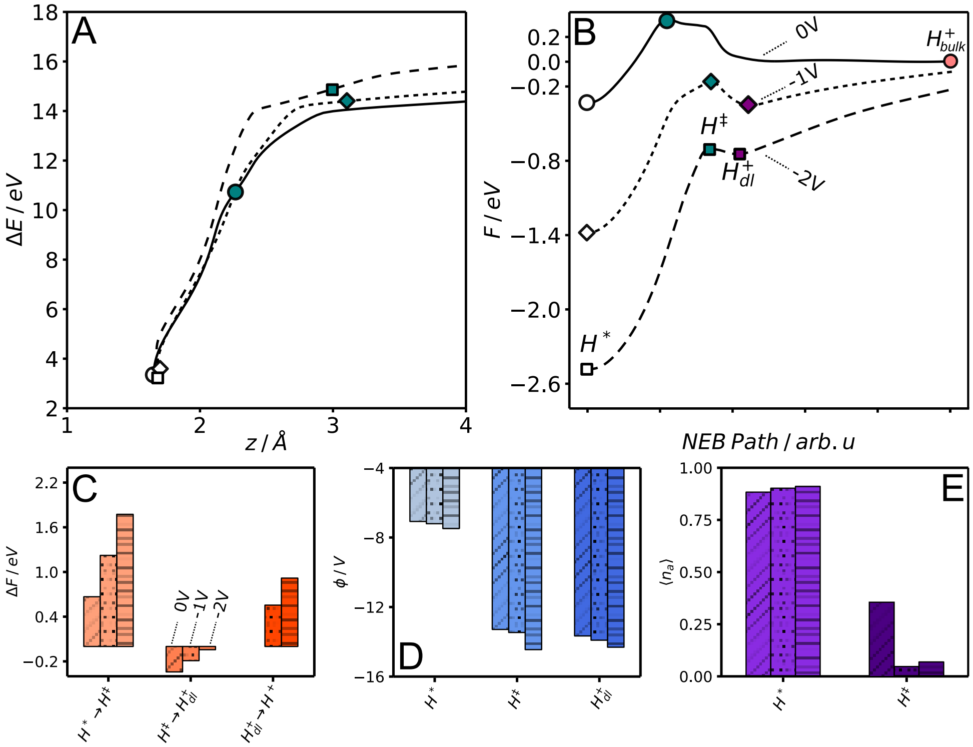

Figure S6. Raising the Fermi level biases the reaction to reduction. 2D adiabatic surfaces analysis for H^+/H redox near Pt (111). Here, the electrode surface potential $V - V_{\mathrm{pzc}}$ and the Fermi level are connected and vary together. Three cases are shown: $V - V_{\mathrm{pzc}} = 0$ V with $\epsilon_a - \epsilon_f = -10.2$ eV, $V - V_{\mathrm{pzc}} = -1$ V with $\epsilon_a - \epsilon_f = -11.2$ eV, and $V - V_{\mathrm{pzc}} = -2$ V with $\epsilon_a - \epsilon_f = -12.2$ eV. **A)** the MFEP along the reaction coordinates ΔE and z for the three electrode potentials. **B)** the free energies along the MFEP projected onto 1D. **C)** the free energy changes for the steps along the MFEP are shown. **D)** the average electrostatic potential experienced by hydrogen is shown for H^*, $H^{\ddagger}$, and H^+_{dl}. **E)** the average expected occupancy is shown for H^* and for $H^{\ddagger}$. (In A and B, the solid, dotted, and dashed lines correspond to the 0 V, -1 V, and -2 V cases, respectively. In C, D, and E, the diagonal bar hatches, dotted hatches, and horizontal bar hatches correspond to the 0 V, -1 V, and -2 V cases, respectively.)

Supplementary Note 8.

Hydrogen – water coordination numbers for adiabatic ET. We calculate the coordination of water atoms around the H atom at various z distances where H is electronically coupled to the surface (Figure S7). The H atom is prepared in its most favorable state at each z (i.e. reduced close to the surface, oxidized far away) and relaxes to equilibrium solvation. As hydrogen approaches the surface and eventually becomes reduced, it loses solvation energy, reflected in the loss of coordinating water oxygens in the first two solvation shells. The first two solvation shells approximately correspond to $r < 1.5$ Å and 1.5 Å $< r < 3.5$ Å, respectively. A stronger surface charge and corresponding interfacial field does not significantly affect the hydrogen solvation structure, and only minor changes in the coordination number profiles are noted (S7A-D).

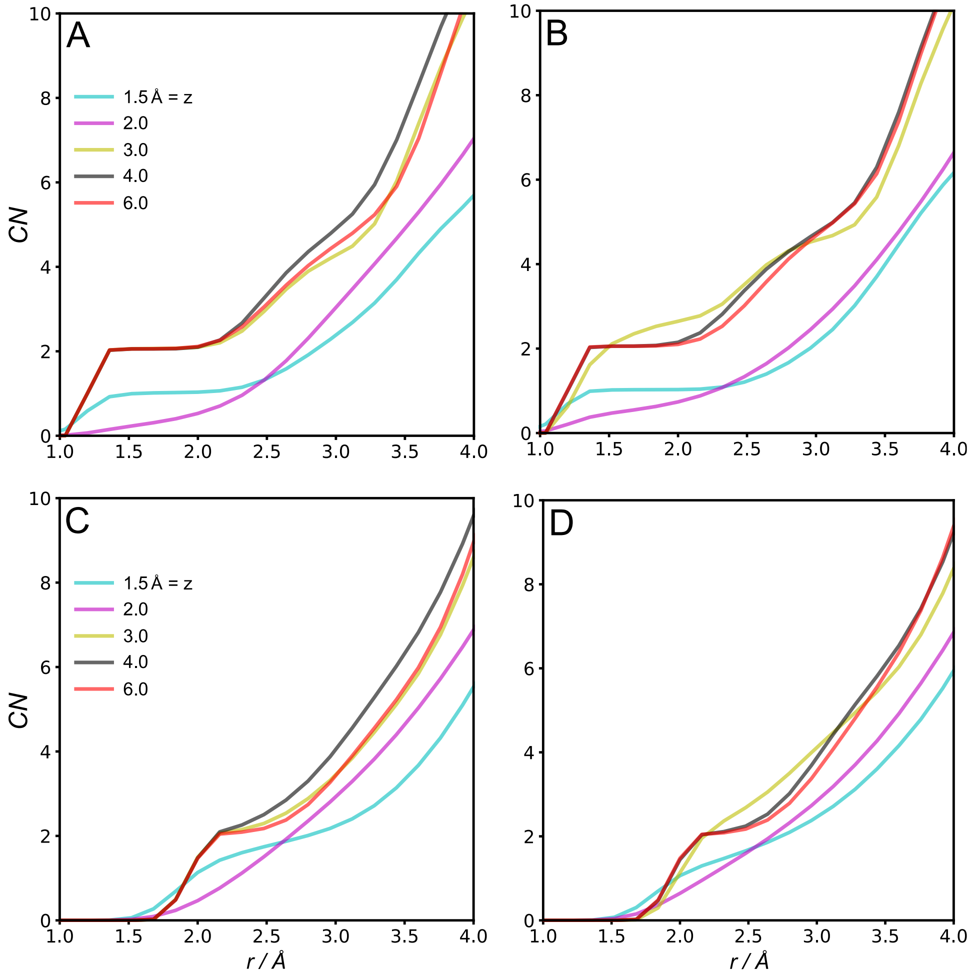

Figure S7. Coordination numbers of water atoms around the redox hydrogen for adiabatic ET at fixed hydrogen z and varied cell voltage $\mathbf{V - V_{pzc}}$. The Fermi level is set to $e_a - e_f = -10.2$ eV. At $z > 2.0$ Å, hydrogen is oxidized, and at $z \leq 2.0$ Å, hydrogen is reduced. Changing $V - V_{\mathrm{pzc}}$ only slightly affects the equilibrium solvation of hydrogen. A) CNs of water O around redox H at $V - V_{\mathrm{pzc}} = 0$ V. B) CNs of water O around redox H at $V - V_{\mathrm{pzc}} = -2$ V. C) CNs of water H around redox H at $V - V_{\mathrm{pzc}} = 0\ V$. D) CNs of water H around redox H at $V - V_{\mathrm{pzc}} = -2$ V.

Supplementary Note 9.

Referencing procedure for proton free energies. Here, we outline a procedure for connecting the free energy scales of simulations performed at different electrode potentials. First, we must pick a reference state. In electrochemical experiments at constant potential, adsorbed hydrogen reaches an equilibrium with H^+ in bulk solution. H^+ in bulk solution is not influenced by the surface, so choosing bulk H^+ as a reference state is convenient. We can simulate a proton in solution in our system, which additionally makes it a convenient choice for a reference. However, we simulate a small capacitor cell, and we must be careful to consider the electrostatics of ion-surface interactions since the Coulomb interactions are long range.

In the simulation, H^+ reaches bulk solvation far from the double layer, and its free energy can be expressed as follows relative to vacuum:

$$F_{H^+} = F_{H^+}^{\mathrm{sol}} + zF\phi_{H^+}$$

where ϕ_{H^+} is the local electrostatic potential at the H^+ position, $F_{H^+}^{\mathrm{sol}}$ is the bulk solvation free energy, and z is the proton charge. In these simulations with water and no electrolyte salt, the Debye length is longer than the cell length. Consequently, when applying a potential difference $\Delta V \neq 0\ V$, the proton is influenced by the surface potential at all points in the cell. However, at $\Delta V = 0\ V$, both electrodes are at the PZC, and the proton at the center of the cell is unaffected by any excess surface charge. Thus, we choose the proton in the center of the cell at $\Delta V = 0 V$ as the reference state, which approximates the bulk proton not influenced by surface charge:

$$F_{H^+}\left(\Delta V = 0, z = \frac{D}{2}\right) = 0$$

For our simulation cell, setting $\Delta V = 1$ V creates an electric field near the middle of the cell on the order of 0.01 V Å^{-1}, small enough to neglect entropic effects from minor changes in bulk structure of water and the solvation shell of the proton. Thus, the only significant change in free energy of the proton is due to the electrostatic potential:

$$F_{H^+}(\Delta V = v, z) - F_{H^+}(\Delta V = 0, z) = zF(\phi_{H^+}(\Delta V = v, z) - \phi_{H^+}(\Delta V = 0, z))$$

In the simulation, the total electrostatic potential of the solvated proton can be decomposed as follows:

$$\phi'_{H^+,tot}(\Delta \mathrm{V}, \mathrm{z}) = \phi'_{sol} + \phi'_{img}(z) + \phi'_{\sigma}(\Delta V, z)$$

Where ϕ'_{sol} is the contribution from the solvation of the proton, ϕ'_{img} is the contribution from primary image charges, and ϕ'_{σ} is the contribution from the higher-order images and excess surface charge on the metal electrodes. We use the prime notation to indicate a Madelung potential is used. Since ϕ'_{img} varies with z, we connect the free energies for two simulations at constant z. Choosing $\mathrm{z} = \mathrm{D}/2 = 25$ Å, we can calculate the proton free energy change when the potential difference $\Delta V = v$ is switched on:

$$F_{H^+}\left(\Delta V = v, z = \frac{D}{2}\right) - F_{H^+}(\Delta V = 0, z = \frac{D}{2}) = zF(\langle\phi'_{H^+,tot}\left(\Delta V = v, z = \frac{D}{2}\right)\rangle - \langle\phi'_{H^+,tot}\left(\Delta V = 0, z = \frac{D}{2}\right)\rangle)$$

Where the ensemble average of $\phi'_{\mathrm{H^+,tot}}$ is performed for a solvated proton at fixed z.

When we perform importance sampling and obtain free energy surfaces as a function of ΔE and z, we can similarly establish a common energy scale. Given two free energy surfaces $F^{(1)}_{H^+}(\Delta E, z)$ and $F^{(2)}_{H^+}(\Delta E, z)$ for ET calculations performed at different cell voltages, we can connect the energy scales across voltages by the following:

$$F^{(2)}_{H^+}\left(\Delta E = \Delta e_{\min}, z = \frac{D}{2},\right) - F^{(1)}_{H^+}\left(\Delta E = \Delta e_{\min}, z = \frac{D}{2},\right)$$
$$= zF(\langle \phi'_{H^+,tot}\left(\Delta V = v, z = \frac{D}{2}\right)\rangle - \langle \phi'_{H^+,tot}\left(\Delta V = 0, z = \frac{D}{2}\right)\rangle)$$

Where the connection is performed at $\Delta e_{\min}$, which is the solvent coordinate value corresponding to equilibrium solvation for the proton. Similarly, the statistics collected for ϕ'_{H^+} are at equilibrium solvation, i.e., no harmonic restraint with respect to ΔE. In our results, we have used the connection formula to establish a common reference for all cell voltages evaluated.

Supplementary Note 10.

Coordination Numbers of water around H‡. From our adiabatic free energy calculations for the Volmer step, we see that the reduction transition state occurs later, i.e., at greater solvent polarization, when the Pt surface is negatively charged. Here, we quantify the change in the solvation structure at the transition state by plotting coordination numbers (CNs) of waters solvating H‡. In Figure S8, the CNs for both water-hydrogens and water-oxygens are shown. The CNs are calculated by de-biasing statistics from a single biased simulation where the proton is harmonically restrained in ΔE and z to the saddle point on the adiabatic free energy surface.

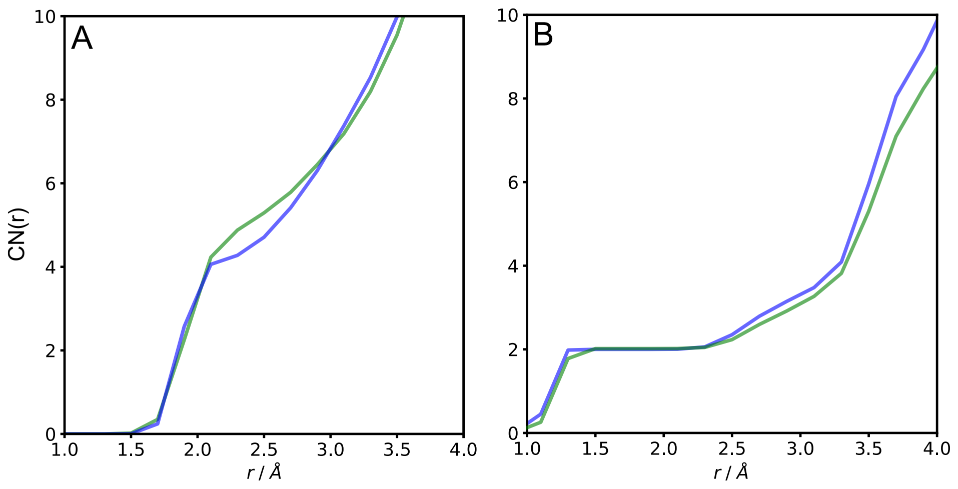

Figure S8. Coordination numbers (CN) of water atoms around hydrogen at the redox transition state from adiabatic simulations. Here we set $\epsilon_a - \epsilon_f = -10.2$ eV. A) H‡ / water-hydrogen CNs. B) H‡ / water-oxygen CNs. (Blue: Electrode at $V - V_{\text{pzc}} = 0$ V, Green: $V - V_{\text{pzc}} = -2$ V.)

At $V - V_{\text{pzc}} = -2$ V, there is higher water-H density near H‡ around r = 2.5 Å and slightly lower water-O density in the 2nd layer of solvating waters, r > 2.5 Å. Since H‡ has a partial positive charge, the change in CNs imply less attractive H‡/water interactions, thus the solvent is polarized further away from equilibrium solvation at the negative voltage transition state.

References

1 Marcus, R. A. On the Theory of Electron-Transfer Reactions. VI. Unified Treatment for Homogeneous and Electrode Reactions. *The Journal of Chemical Physics* **43**, 679-701, doi:10.1063/1.1696792 (1965).

2 Newns, D. M. Self-Consistent Model of Hydrogen Chemisorption. *Physical Review* **178**, 1123-1135, doi:10.1103/PhysRev.178.1123 (1969).

3 Santos, E., Lundin, A., Pötting, K., Quaino, P. & Schmickler, W. Model for the electrocatalysis of hydrogen evolution. *Physical Review B* **79**, doi:10.1103/PhysRevB.79.235436 (2009).

4 Straus, J. B., Calhoun, A. & Voth, G. A. Calculation of solvent free energies for heterogeneous electron transfer at the water–metal interface: Classical versus quantum behavior. *The Journal of Chemical Physics* **102**, 529-539, doi:10.1063/1.469431 (1995).

5 Lam, Y. C., Soudackov, A. V., Goldsmith, Z. K. & Hammes-Schiffer, S. Theory of Proton Discharge on Metal Electrodes: Electronically Adiabatic Model. *J Phys Chem C* **123**, 12335-12345, doi:10.1021/acs.jpcc.9b02148 (2019).

6 Huang, J. Mixed quantum-classical treatment of electron transfer at electrocatalytic interfaces: Theoretical framework and conceptual analysis. *J Chem Phys* **153**, 164707, doi:10.1063/5.0009582 (2020).

7 Petersen, M. K., Kumar, R., White, H. S. & Voth, G. A. A Computationally Efficient Treatment of Polarizable Electrochemical Cells Held at a Constant Potential. *J Phys Chem C* **116**, 4903-4912, doi:10.1021/jp210252g (2012).

8 Santos, E., Lundin, A., Potting, K., Quaino, P. & Schmickler, W. Model for the electrocatalysis of hydrogen evolution. *Physical Review B* **79**, doi:10.1103/PhysRevB.79.235436z (2009).

9 Mejías, J. A. & Lago, S. Calculation of the absolute hydration enthalpy and free energy of H+ and OH−. *The Journal of Chemical Physics* **113**, 7306-7316, doi:10.1063/1.1313793 (2000).

10 Calhoun, A., Koper, M. T. M. & Voth, G. A. Large-scale computer simulation of an electrochemical bond-breaking reaction. *Chemical Physics Letters* **305**, 94-100, doi:10.1016/s0009-2614(99)00353-x (1999).

11 Le, J. B., Fan, Q. Y., Li, J. Q. & Cheng, J. Molecular origin of negative component of Helmholtz capacitance at electrified Pt(111)/water interface. *Sci Adv* **6**, doi:10.1126/sciadv.abb1219 (2020).

12 Li, C. Y. *et al.* In situ probing electrified interfacial water structures at atomically flat surfaces. *Nat Mater* **18**, 697-701, doi:10.1038/s41563-019-0356-x (2019).

13 Maroncelli, M. & Fleming, G. R. Computer simulation of the dynamics of aqueous solvation. *The Journal of Chemical Physics* **89**, 5044-5069, doi:10.1063/1.455649 (1988).

14 Hynes, J. T. Chemical reaction rates and solvent friction. *Journal of Statistical Physics* **42**, 149-168, doi:10.1007/bf01010844 (1986).
